# Supplementary material for: Life cycle assessment needs predictive spatial modelling for biodiversity and ecosystem services
Source: Nat Commun. 2017 Apr 21;8:15065. doi: 10.1038/ncomms15065 (PMC5413941; doi:10.1038/ncomms15065)
Supplement: Supplementary Information — Supplementary Figures, Supplementary Tables, Supplementary Notes, Supplementary Methods and Supplementary References [file ncomms15065-s1.pdf]

Supplementary Methods for Chaplin-Kramer et al. Life Cycle Assessment Needs Predictive, Spatial Modelling for Biodiversity and Ecosystem Services

In order to estimate the environmental impacts of different feedstocks and locations, and to ascertain the degree to which spatially explicit data and methods change the results, we conduct an LCA for high density polyethylene (HDPE) bioplastic using two alternative approaches. After defining the demand and therefore feedstock production scenarios (Section 1), we first conduct a standard attributional, ISO4040 compliant LCA (hereafter "standard LCA"; Section 2), calculating land-use change (transformation) impacts with the direct land-use change assessment tool developed by Blonk Consultants (Section 2.4.2). This represents historical changes in the land cover for the given crops (sugarcane and maize) and country of production (Brazil and USA) over the last 20 years. Second, we develop a "land-use-change-improved" (LUCI) approach to LCA (Section 3), in which we use the results of spatially-explicit modelling of land change and its impacts on biodiversity and ecosystem services to inform the agricultural stage of the standard LCA. To illustrate the impact of scale on sourcing decisions, we apply each approach to a range of demand scenarios for bioplastic and therefore crop production and the land-use changes to meet them.

The LUCI-LCA is based on attributional inventories but considers forward-looking expansion and intensification on new land, based on predictive spatial modelling to meet demand for the new material (see Supplementary Note 1 for definitions of attributional compared to consequential LCA). We assume the new demand is additional to maintaining existing agricultural production levels for current uses. The modelling takes into account historical trends for both intensification of production on existing land and suitability of land for expansion in the area. Below, we discuss the various elements of our approach: 1) definition of demand scenarios, 2) elements of the standard LCA, 3) elements of LUCI-LCA approach, including predictive land change modelling, biodiversity and ecosystem service modeling, and 4) integration of LUCI into LCA, including adaption of existing life cycle inventory (LCI) used in the standard LCA.

1. Agricultural crop demand scenarios

In this use case, we apply conversion pathways from the literature (as given in Supplementary Figure 1) to determine how much raw material (sugarcane or maize) is required to meet the HDPE demand scenarios. The volumes of feedstock given in Supplementary Table 1 correspond to differing bio-HDPE demand scenarios. However, recognizing the importance of geographical influences on the results of such assessments, two different feedstocks in two locations are considered to demonstrate the new LUCI-LCA approach. Different scenarios are also informed by an understanding that impacts could differ with greater volume requirements due to the different spatial patterns of land-use change that result (Chaplin-Kramer et al.[1]). For this reason, the first two volume scenarios are set at the largest scales that could be induced directly by Unilever, with a subsequent scenario set to represent broader sectorial uptake of the bio-HDPE.

Supplementary Table 1. Scenarios considered (metric tonnes of HDPE plastic, source of feedstock and relative feedstock volume)

Market	HDPE amount (T)	Feedstock* amount (T)	
1. Unilever's use of HDPE North America (2012)	23,000	Maize USA 134,140	Sugarcane Brazil 615,278
2. Unilever's total use of all plastics in North America (2012)	86,000	Maize USA 501,567	Sugarcane Brazil 2,300,603
3. Extrapolated volume North America	321,000	Maize USA 1,872,130	Sugarcane Brazil 8,587,134

* based on sugarcane fresh matter and dried maize grains

The scenarios considered include:

1. Unilever's approximate HDPE packaging volume used in North America in 2012 and assumed to be met either from maize grown in the USA or sugarcane from Brazil.
2. Unilever's approximate total plastic packaging volume (all plastics, i.e. PP+PET+HDPE) used in North America in 2012. This total volume was considered as HDPE in the case study to give a sense of the impacts that would emerge if they were sourced from bio-feedstocks; again they were assumed to be met either from maize grown in the USA or sugarcane from Brazil.
3. An extrapolated volume included to scale the demand through the sector (calculated by taking the ratio of Unilever HDPE volume and Unilever plastic volume in North America and multiplying by the Unilever plastic volume in North America) met either from maize grown in the USA or sugarcane from Brazil.

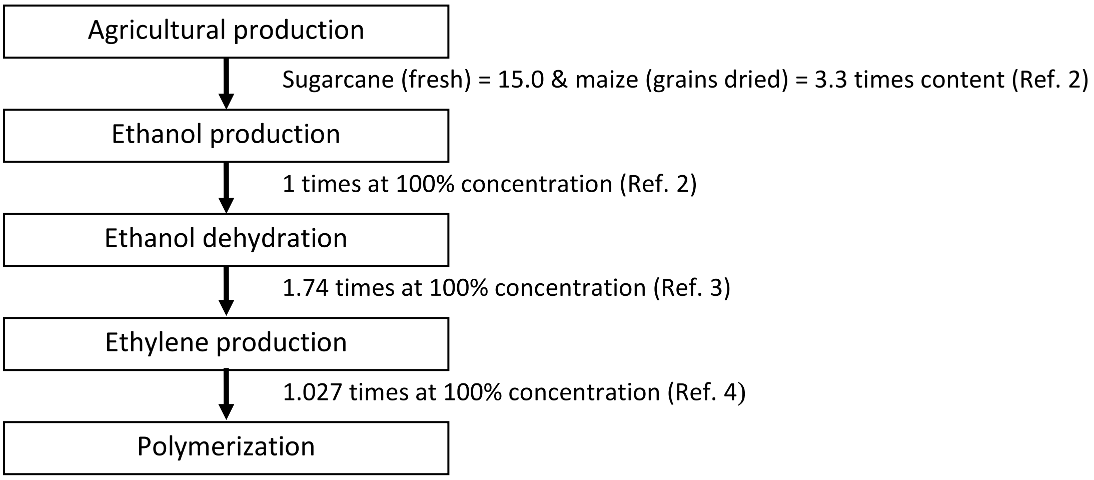

Supplementary Figure 1. Production flow diagram and conversion rates for sugarcane and maize, with references[2, 3, 4]

2. Standard Life Cycle Assessment Methodological Details

2.1. System boundary

The system boundary for the LCA is presented in Supplementary Figure 2. The analysis includes the production of agricultural raw material with all the associated inputs and emissions as well as land use change, ethanol production and dehydration, ethylene production and polymerization. All relevant transport steps are considered. Ethanol production is considered to occur at the same place as dehydration; ethylene production occurs at the same place as polymerization. As there are no differences in consumer use for HDPE derived from different feedstocks, the consumer use phase is excluded from the analysis. The end-of-life emissions are considered in a simplified manner: all the carbon in the bio-HDPE is assumed to be released at the end of the product life as carbon dioxide (CO_2) with no contribution towards Global Warming Potential, since the CO2 released was sequestered in crop growth.

We highlight here that for the impact categories Biodiversity Damage Potential, Erosion Potential and Water Consumption, impact assessment is limited to the agricultural stage. This is because the land area occupied by other life cycle stages is relatively small, compared to the agricultural production stage, and land use and land use change is the main driver of these impact categories. Global Warming Potential and Eutrophication Potential are considered at each stage of the life cycle within the full system boundary.

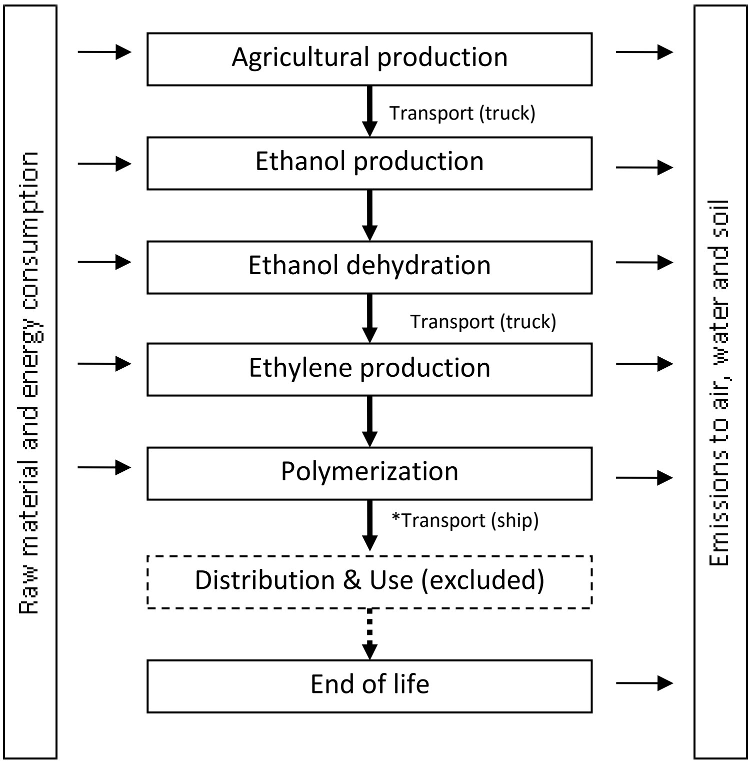

Supplementary Figure 2. System boundary for the production of bio-HDPE (*transport of sugarcane-based HDPE from Brazil to USA).

2.2. Allocation

Economic allocation is applied to allocate inputs and emissions to the main product and any co-products: distiller's dried grains with solubles during maize ethanol production and electricity during sugarcane ethanol production, following the default approach in the ecoinvent database 2.2.

2.3. Impact Assessment Methodology

The following impact categories and methods are used:

- Global warming potential (GWP) - IPCC AR5[5] 100 years excluding biogenic carbon method. The GWP associated with land use change (LUC) is considered as given in section 2.4.2
- Marine eutrophication potential – ReCiPe (Goedkoop et al.[6])
- Biodiversity damage potential –Mean Species Abundance (MSA; GLOBIO[7]), according to de Baan et al.[8] The MSA observations of selected pressure factors for 'Conventional farming' and 'Perennial tree crop' are chosen for the land use and land use change areas for annual cropland and perennial cropland respectively in the assessment of biodiversity damage potential. For land use change from forest, an MSA factor of 1 is used.
- Erosion regulation potential (ERP) – Saad et al.[9]
- Consumed water - No impact assessment performed for main results, the indicator used is water consumption (Mekonnen et al.[10]); impact assessment performed with AWARE method in sensitivity analysis (see Section 3.3.3)

2.4. Life Cycle Inventory

2.4.1. Agricultural production

The inputs and emissions for the production of sugarcane in Brazil and maize in the USA are obtained from the ecoinvent 2.2 datasets 'BR: sugar cane, at farm' and 'US: corn, at farm' (Ref. 2) respectively and the inventories are revised to take into account updated crop irrigation figures (Supplementary Table 2). The volume of consumed (or blue) water for irrigation is derived from the Water Footprint Network (WFN) database (Ref. 10). Irrigation efficiency factors are taken from Rohwer et al.[11] The ecoinvent 2.2 dataset 'US: irrigating'[12] is used to model the irrigation of sugarcane, but the US electricity mix is replaced with a Brazilian one. In the maize system, we also replace the inventory 'CH: irrigating' with the 'US: irrigating'. These values for irrigation correspond to other sources (see 3.2.3.3.1 and 3.2.3.3.2).

Supplementary Table 2. Updated volumes of irrigation water based on WFN (Ref. 10) and Rohwer et al. (Ref. 11)

Crop	Consumed volume of water for irrigation per T crop [m^3T^{-1}] (WFN)	Irrigation efficiency (Ref. 11)	Irrigation blue water volume consumption per T crop [m^3T^{-1}]
Sugarcane in Mato Grosso, Brazil	0.32	0.55	0.58
Maize in Iowa, USA	1.65	0.55	3.01

2.4.2. Land-Use Change (LUC)

The amount of land use change (transformation) and carbon dioxide emissions resulting from land transformation are estimated using the direct land use change assessment tool (Blonk Consultants 2014)[13], following the standards PAS2050-1[14], Greenhouse Gas (GHG) Protocol[15] and EnviFood Protocol[16] – "country known, previous land use unknown" situation

The area of land transformation used in the assessment of Erosion Potential and Biodiversity Damage Potential are given below (Supplementary Table 3) and the GHG emissions from land transformation for sugarcane and maize are 10.81 T CO_2eq/ha*year and 0.02 T CO_2eq/ha*year respectively. The figures for both the area of land transformation and GHG emissions from land transformation are derived in the tool as annual figures).

Supplementary Table 3. Land transformation amortized over 20 years derived from the Direct Land Use Change Assessment Tool (Ref. 13), weighted average approach (Version 2014.1, published January 2014)

Land transformation type	Sugar cane in Brazil	Maize in the United States
	m^2 ha^{-1}	m^2 ha^{-1}
From Forest (average) to Annual cropland	188	0
From Grassland (average) to Annual cropland	0	0
From Perennial cropland to Annual cropland	13	1
From Annual cropland to Annual cropland	70	75
No transformation attributed	9730	9924

2.4.3. Ethanol Production

The resource use and emissions for the production of ethanol are obtained from the ecoinvent 2.2 datasets 'BR: ethanol, 95% in H2O, from sugar cane, at fermentation plant' and 'US: ethanol, 95% in H2O, from corn, at distillery' respectively. These are representative of ethanol production in Brazil and the United States (Ref. 2).

2.4.4. Ethanol Dehydration

The dehydration of hydrated ethanol 95% to anhydrous ethanol is considered using the datasets 'BR: ethanol, 99.7% in H_2O, from biomass, at distillation' and 'US: ethanol, 99.7% in H2O, from biomass, at distillation'. The Brazilian ethanol dataset considers the production from both sugarcane and molasses (by-product from sugar production). For the purposes of this study, we assume that 100% of new ethanol is produced from new sugarcane. We adjust the ecoinvent dataset to consider 100% production from sugarcane.

2.4.5. Ethylene Production

The LCI for the production of ethylene from ethanol is based on the processing requirements given in Kochar et al. (Ref. 3) shown in Supplementary Table 4. The steam is modelled using the dataset 'RER: steam, for chemical processes, at plant'[17], power is modelled using the ecoinvent country specific electricity mix 'BR: electricity, high voltage, production BR, at grid'[18] and 'US: electricity, medium voltage, at grid' for Brazil and USA respectively, and fuel is assumed to be gas and modelled using the ecoinvent dataset 'RER: natural gas, burned in industrial furnace >100kW'[19].

Supplementary Table 4. Inputs required for the production of polymer grade ethylene (Ref. 3)

	Amount	Unit
Ethanol (100% concentration)	1.74	t t^{-1} ethylene
Steam, from natural gas	1.21	t t^{-1} ethylene
Electricity	1.12	GJ t^{-1} ethylene
Fuel (net)	1.68	GJ t^{-1} ethylene

2.4.6. Polymerization

The polymerization of ethylene to HDPE is modeled following the approach of Tsiropoulos et al. (Ref. 4). Monomer consumption in kilograms per metric tonne of product is 1027 kg/T (according to the European Commission[20]).

2.4.7. Transport

Transportation is modeled for relevant life cycle phases where it is not already included in the existing datasets. Transport includes the trucking of product from the ethanol production plant to the ethylene production plant (500 km by road for both regions) and the shipping of HDPE produced from sugarcane in Brazil to the US market (10,100 km according to Sea-Distances.org[21]). Transport is modelled using ecoinvent datasets 'RER: transport, lorry >16t, fleet average' and 'OCE: transport, transoceanic freight ship'.[22]

2.4.8. End-of-Life

All the carbon stored in the bio-HDPE is assumed to be released back to the atmosphere at the end of the product life in the form of carbon dioxide (CO_2) with no contribution towards global warming potential, since the CO2 released was sequestered in crop growth.

2.5. Sensitivity analysis

A sensitivity analysis is provided for scenario 3 (321,000 T HDPE). The effects of pumping water for irrigation are varied using lower and upper irrigation water volumes based on the lower and upper consumed water volumes (Supplementary Table 5) considering irrigation efficiencies (as given in Supplementary Table 2). Similarly, the N-fertilizer application rates and yields are varied using the lower and upper N-fertilizer application rates (as given in Supplementary Table 6). These changes affected estimates for Global Warming Potential and Eutrophication Potential. For Biodiversity Damage Potential and Erosion Potential, the yield changes associated with the upper and lower N-fertilizer application rates are applied and for the Biodiversity Damage Potential combined with the lower and upper MSA (Mean Species Abundance) values from GLOBIO (Supplementary Table 7). For Water Consumption, the yield changes associated with the upper and lower N-fertilizer application rates are applied and combined with the lower and upper consumed water values as estimated using the LUCI-LCA values; the relative differences in consumed water from the base case in the LUCI-LCA were applied to the LCA base case.

Supplementary Table 5. Consumed water (m^3/T crop) for sugarcane (Mato Grosso) and maize (Iowa)

	Base case	Lower	Upper
Sugarcane (LCA)	0.32	0.08	0.40
Maize (LCA)	1.65	0.31	2.79

Supplementary Table 6. Changes in nitrogen application rates (%) and crop yields considered in sensitivity analysis used in LCA

	Change in nitrogen application rate		Change in yield	
	Lower	Upper	Lower	Upper
Sugarcane	-45%	+45%	+9%	-9%
Maize	-15%	+15%	+9%	-9%

Supplementary Table 7. Sensitivity analysis for Biodiversity Damage Potential (GLOBIO)

	Base case	Lower	Upper
Conventional farming	0.84	0.75	0.93
Perennial tree crop	0.78	0.64	0.92

3. Land Use Change Improved (LUCI) Life Cycle Assessment Methodological Details

To produce the LUCI-LCA, we first develop predictive land change models (LCM) to translate the demand scenarios into maps of agricultural expansion and intensification (section 3.1). We feed the resulting land-use change maps into models for biodiversity and ecosystem services (InVEST) in order to assess the environmental impacts of the additional product demand in a spatially explicit way (section 3.2). Finally, we integrate the results to substitute for key elements of the land-use change impacts in standard LCA, as illustrated in Fig. 1 in the main text (and described in detail in section 3.3).

3.1. Land Use Change Modeling to Spatialize Demand Scenarios

This section summarizes the process for generating spatial scenarios of agricultural expansion and intensification resulting from changes in commodity demand in a region. This approach can be applied with public, globally available data and limited land change modeling expertise. The approach we developed has three steps, described in detail below:

1. Derive the potential land area for expansion required to achieve the increase in production based on expansion only
2. Adjust the total land expansion to account for intensification by creating spatially explicit yield map to partition production into amounts met through expansion and intensification
3. Allocate the expansion area spatially within the region of interest

3.1.1. Derive the area of expansion

The potential expansion area is derived through an extrapolation of past trends in production and the harvested crop area. In order to allow for a globally replicable approach that can be used across supply chains, we prioritized globally-available data to maintain consistency between study regions. We use national FAO data[23] to relate production and area of a particular crop over a time series, regressing production in each year against harvest area in each year. The slope of this line provides a more appropriate estimate of agricultural expansion to meet a production target than yields alone because it incorporates past gains through both intensification and expansion. If past production from one year to the next has increased more than can be accounted for by multiplying past increases in harvested area by yield in the first year, then the remaining

production must be attributable to intensification. If yields are used instead of this time series relationship (i.e., dividing production target by current yields), the total area predicted for expansion could be higher than is likely to occur in reality because no intensification would be assumed. Since the slope of the regression we are using is the expected increase in production for an expected increase in area, the production target is simply divided by the regression slope in order to solve for the area of expansion needed. We are assuming the rate of intensification will continue on its previous trajectory.

To illustrate this in the context of our use case, the solid line in Supplementary Figure 3 represents the regression; the production increase per area increase based on past trends, which reflects a mixture of intensification and expansion. The dotted line represents the expansion required if there is no intensification, if current yields are applied to reach production target (i.e., the slope of the dotted line is the most recent yield or average yield from the past several years if highly fluctuating).

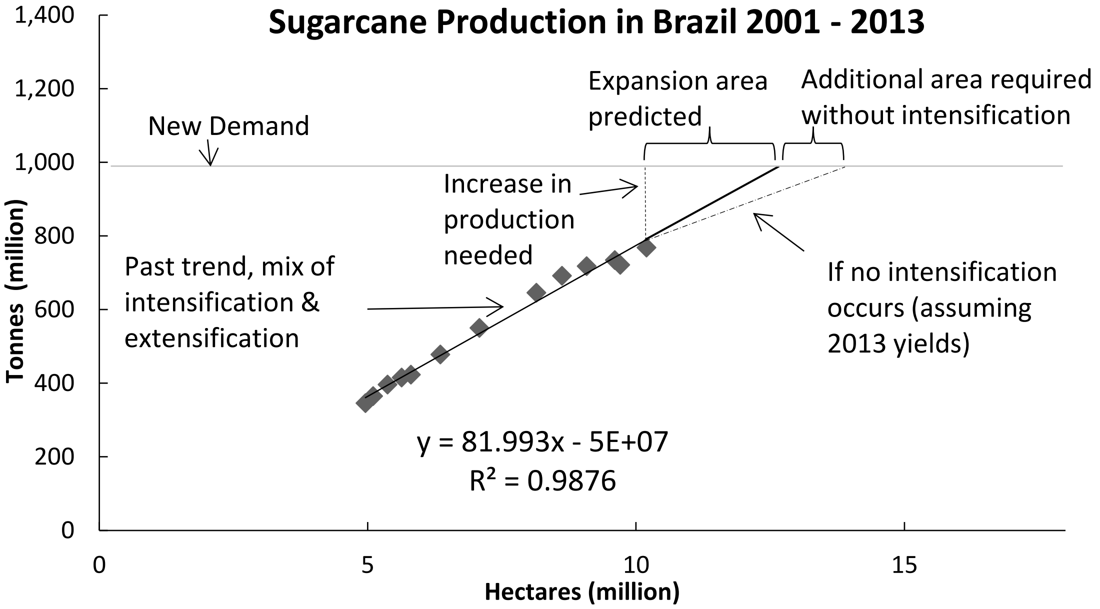

Supplementary Figure 3. Case study example of regression to derive area of expansion from past trends on production and harvested area.

In this case study, Mato Grosso shows evidence of intensification based on past trends, whereas Iowa does not. That is, changes in production in recent years can be attributed solely to changes in harvested area in Iowa. In Brazil we find the slope of the regression between production and area to be 81.9 (y = 81.993x – 5E07); thus for every 82 metric tonnes of sugarcane production, 1 hectare of land was converted to sugarcane production in Brazil. In order to calculate the expansion area predicted by past trends for each demand scenario, then, the production target is divided by the slope of this regression (Supplementary Table 8).

Supplementary Table 8. Production targets and area of expansion predicted for each demand scenario

Study area	Scenario	Crop	HDPE demand (T)	Conversion Factor	Crop production target (T):	Area of expansion predicted (ha):
MT	1	Sugarcane	23,000	26.75	615,278	7,504
MT	2	Sugarcane	86,000		2,300,603	28,059
MT	3	Sugarcane	321,000		8,587,134	104,730
IA	1	Maize	23,000	5.83	134,140	12,008
IA	2	Maize	86,000		501,567	44,898
IA	3	Maize	321,000		1,872,130	167,584

3.1.2. Partition production into expansion and intensification

To derive the amount of crop produced through expansion, average yields for the most recent year (Ref. 23) are multiplied by the expanded area (determined in 3.1.1). This value is then subtracted from the demand target to derive the amount of commodity produced through intensification (Supplementary Table 9). If, in the last 10 years of production, increases can be attributed solely to an increase in harvested area (as was the case for Iowa; meaning the slope of the line determined in 3.1.1, was the same as the current yield, 11.1 T/ha), the amount of production achieved through expansion should be equal to the demand target.

For simplicity, the yield increase for intensification is specified as exactly that required for all of the target production to occur on the land predicted for agricultural expansion. The area of intensification is therefore equal to the area of expansion, and the intensified yield is calculated as the slope of the line presented in 3.1.1. In the case of Brazil, this requires a yield of 82 T/ha for all scenarios (up from a current average yield of 75.3 T/ha). Thus, according to this estimate, the amount of increased production met through intensification is less than 10% of the total increase. The effects of intensification are assumed to apply to all of the sugarcane expansion area. This is not necessarily a realistic prediction (i.e., yields could be expected to rise both in current and future sugarcane production areas, not exclusively in, and evenly across, all future sugarcane production). However, it is not possible with currently globally available data to spatially attribute past intensification, because maps of fertilizer use and crop-specific land-uses are not available at the resolution necessary for our ecosystem services modeling (500 m or less).

Supplementary Table 9. Production and area predictions for expansion and intensification of sugarcane in Mato Grosso, Brazil (with an intensified yield of 82 T/ha up from 75.3 T/ha average current yield; still well within the achievable yields for this climate[24]), and expansion of maize in Iowa, USA (with average current yield of 11.1 T/ha).

Region, Scenario	Production target (T):	Area of expansion (ha):	Expansion production (T)	Intensification production (T)
MT 1	615,278	7,504	575,438	39,840
MT 2	2,300,603	28,059	2,143,381	157,222
MT 3	8,587,134	104,730	7,996,665	590,469
IA 1	134,140	12,008	135,263	-
IA 2	501,567	44,898	501,567	-
IA 3	1,872,130	167,584	1,872,130	-

3.1.3. Spatially allocate the expansion area

Our approach for spatially allocating the agricultural expansion required to meet the production target involves creating a suitability layer that assigns eligible pixels an index value which reflects biophysical suitability for conversion to agriculture. Specifically, the approach relies on estimating suitability using a logistic regression where the 0/1 dependent variable indicates whether a pixel is classified as agriculture in a particular year. After regressing the dependent variable against multiple driver variables on a subset of the data, the suitability layer is generated by interpreting predicted probabilities from the logistic regression as a suitability value for each pixel capable of conversion to agriculture. These values are then sorted, with the pixels that have the highest ranking values chosen for conversion to agriculture until the area requirements of the demand scenario have been met.[25]

This approach makes the fundamental assumption that factors that have historically determined the location of agriculture will also determine the location of agricultural expansion driven by increases in demand for the commodities specified in the demand scenario (i.e. in our case, maize and sugarcane). Part of this assumption is related to the static nature of the regression: that is, we predict where agriculture exists at a point in time. The other part of this assumption is due to coarseness in the representation of agriculture: we are unable to identify datasets with global coverage that resolve agriculture at more refined classifications (i.e. crop species

or categories of crop). Therefore, we are unable to account for the fact that expansion of certain crops may be driven by different variables than those that drive "agriculture" generally. The benefit of taking this general agriculture perspective is that we are representing total land-use change, which includes not only the direct land-use change that results from a particular crop replacing natural habitat, but also a proxy for indirect land-use change that results from one crop displacing another crop that ultimately causes conversion of natural habitat (indirect land-use change). That is, it will likely be the case that meeting a demand scenario will not cause conversion of natural habitat to farming of the desired feedstock at the areas specified in the demand scenarios, since crop-to-crop conversion on farmland may occur. But this crop-to-crop conversion may cause other commodities to drive expansion into natural lands. We believe this makes the LUCI-LCA approach a more conservative (in the sense of assessing worst-case impacts) way to consider the full change that will likely be provoked within the regions of study, whether directly or indirectly.

We do not account for leakage effects outside the geographic boundaries of the study system (in this case, the states of Mato Grosso or Iowa); instead we assume that all commodity demand and associated agricultural expansion will occur within the study regions. This is because the effects of crop displacement are more profound on the local level. It is more likely that the shift from cattle ranching to sugar cane cultivation will lead to deforestation for cattle ranching within the same country and region. The direct link between the shift in types of cultivation in one part of the globe and land use changes in the other parts of the globe is difficult to demonstrate.

3.1.3.1. The Logistic Regression Framework

Land cover (MODIS[26]) data from a reference year (in this case, 2007) are reclassified according to binary variables indicating whether each pixel is classified as agriculture. Areas that are assumed to be unable to convert to agriculture (urban, barren, water) are omitted from the regression. Given a set of K driver variables (discussed below), a regression (with slope coefficients β for each driver variable) is then run with the following form:

$$\pi(ag = 1) \sim \beta_1 x_1 + \beta_2 x_2 + \ldots + \beta_K x_K$$

Where π is the logistic transform that translates the probability (p) of the outcome variable taking on a value of unity to a variable suitable for linear regression:[27]

$$\pi = \log \frac{p(x)}{1 - p(x)}$$

The driver variables x_k can be transformed based on theory or empirical knowledge – for example, topography may be an important predictor of suitability for agriculture, but variations in a relatively shallow slope may be unimportant. In this case, we found this to be true, and created a new variable that creates a binary indicator for "shallow" and "steep," based on whether slope is below or above a specific value (see 3.1.3.2).

Logistic regressions are estimated using maximum likelihood estimators, which identify the combination of parameter values that are most likely to produce the observed data. When working with relatively large rasters, the potential for spatial autocorrelation can typically be addressed by fitting the regressions to relatively sparse sample of the rasters (between 1% and 10% in different model runs). This was the approach used for generating the scenarios used for our analysis. Logistic regression can be implemented in most common statistical packages; we used the R package "lulcc" which provides a workflow to connect raster data to the glm function for generalized linear models.

3.1.3.2. Data Sources and Selection of Driver Variables

A key component of this approach is to develop a process that can function with only global data.

Supplementary Table 10 provides an overview of globally available data relevant for modeling agricultural expansion. Additional global data layers of potential relevance could be included (e.g., the Global Roads Open

Access Data Set[28]), but our initial small set is sufficient to represent the types of ecosystem impacts that occurred from historic agricultural expansion (see Fig. 3, main manuscript).

Supplementary Table 10. Data sources considered for land change model

Driver Variable	*Resolution*	*Source*	*Full Name*	*Description*
Bulk Density<br>Cation Exchange Capacity<br>pH<br>Soil Organic Carbon<br>Sand Percent<br>Silt Percent<br>Clay Percent	1km	ISRIC	International Soil Reference Information Center	Group that aggregates soil data from multiple sources into a single 1km resolution product.
Slope	90m	SRTM	Shuttle Radar Topography Mission	NASA mission to collect global elevation data, now available at 30m resolution globally.
Annual Land Cover 2001-2012	500m	MCD12Q1	MODIS Land Cover Data	One of the land cover products from the MODIS missions.
Mean Annual Precip<br>Mean Annual Temp	1km	WorldClim[29]	World Climate Data	Global climate data for ecological modeling

For land cover data, MODIS years 2001, 2007 and 2012 are used as the primary years for exploratory analysis and model testing,[30] with the model ultimately generated using 2007 data to allow validation against datasets from later years (see Section 3.1.3.3). Land cover data specifying agriculture are always required to generate the dependent variable layer, but may also be used to derive alternative driver variables such as distance from the current agricultural frontier or from urban centers. All data are resampled to the 500 meter resolution of the MODIS land cover, using bilinear resampling for continuous data, and nearest neighbor for categorical.

The slope variable was transformed (after resampling) to a binary variable indicating slope as greater than five percent (though testing reveals results are insignificant to thresholding between four and twelve percent). All other variables described in Supplementary Table 11 are untransformed when tested for inclusion in the linear logistic regression. Applying screening tests for correlation using Kramer's V statistic, as well as subsequent inspection of logistic regression results, the final functional forms of the regression (with intercept β_0 and slope coefficients β_x) used for the two states are:

IA: $\pi(ag = 1) = \beta_0 + \beta_1 pH + + \beta_2 ShallowSlope + \beta_3 SiltPct$

MT: $\pi(ag = 1) = \beta_0 + \beta_1 pH + \beta_2 ShallowSlope + + \beta_3 ClayPct + \beta_{24} SoilOrganicCarbon$

The specific values of the model fit are listed in Supplementary Table 11.

Supplementary Table 11. Coefficients of predictor variables in land change model for the study regions.

**Coefficient**	**IA**	**MT**
Intercept	-33.4219	-25.2763
pH	0.2557	0.2489
Shallow Slope	0.5704	2.2446
SiltPct	0.4330	
ClayPct		0.2621
Soil Organic Carbon		-0.2787

3.1.3.3. Model Performance

In the context of our application and data limitations, there is not a single test and metric that provides a good summary of model predictive performance. A first consideration is how well the model predicts the transitions from different types of habitat into agriculture. We considered this by comparing the actual land use change that occurred between 2007 and 2012 in both regions (Iowa for maize and sugarcane from Mato Grosso) with the output from the logistic land change model. This was accomplished by isolating the agricultural expansion land use change only (pixels that switched from non-agricultural non-urban to agricultural) for MODIS 2007 and MODIS 2012 land cover data. Supplementary Figure 4 summarizes the transitions resulting from this land-use change model, classified by habitat type. Very different patterns emerge in the two regions, with maize expansion in Iowa predicted to occur predominantly on forested land, and secondarily on grassland, while sugarcane expansion is predicted to occur almost exclusively on savanna in Mato Grosso. Interestingly, the logistic LCM is more accurate at predicting the proportion of change in different habitats, the larger the change considered. That is, there is a closer match to actual historical changes, in terms of the relative proportions of each type of habitat converted, for scenario 3 with a volume of 321,000 T HDPE than scenario 1 with a volume of 23,000 t. In both regions and for all scenarios, the proportion of each habitat predicted to be converted to agriculture by the logistic LCM matches actual change (red bars in Supplementary Figure 4) much better than the changes assigned in the standard LCA (grey bars). In standard LCA, all habitats for the additional maize and sugarcane production required to reach the demand targets is based on national-level estimates from the previous 20 years (according to the Direct Land Change Assessment tool),[13] which only tracks annual and perennial cropland, grassland, and forest. As previously noted, it assumes much of the land for both crops will come from existing cropland, and in fact nearly all of the "conversion" in Iowa is counted as coming from other crops (which is why no yellow bars appear in the Iowa plot for Supplementary Figure 4).

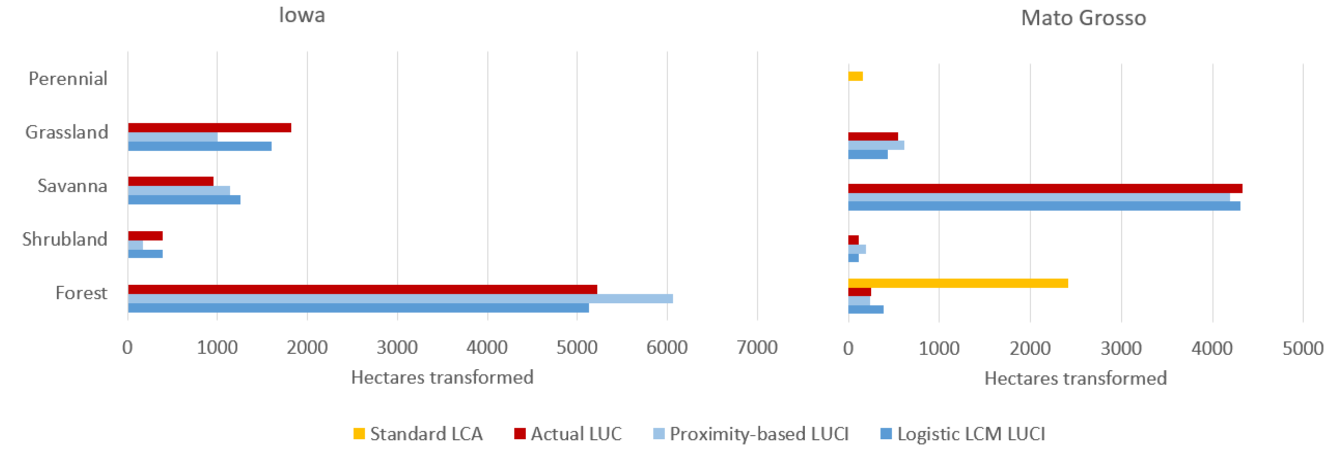

Supplementary Figure 4. Comparison of the habitat types converted for each scenario in the different methods of LCA and in actual agricultural expansion that occurred between 2007-2012 in Iowa and Mato Grosso. Proximity-based LCM and standard LCA LUC shown for scenario 3. Actual agricultural expansion shown for an area equivalent to scenario 3.

For comparison, we also ran the InVEST Scenario Generator: Proximity Based model for the total hectares of expansion predicted for Scenario 3 (320,000 T HDPE), to generate maps of agriculture expanding out from current cropland. This essentially counts the pixels closest to current agriculture as most "suitable". The proximity-based agricultural expansion often matches the actual compositional changes more closely than the logistic model for Mato Grosso, but not for Iowa (light blue bars in Supplementary Figure 4). However, in both regions the proximity to agriculture land change model yields results that more closely align to actual change than those achieved using the Direct Land Use Change Assessment Tool for standard LCA (Ref. 13). The trend depicted in the actual change calculated through MODIS matches that of available agricultural census data (Supplementary Note 2).

Because spatial configuration and not only compositional changes matter to the provision of ecosystem services, we also consider what percent of new agricultural conversion the model correctly predicts between 2007 and 2012. The logistic regression (first row in Supplementary Table 12) correctly predicts conversion to

agriculture (i.e. changes in land cover pixels from 2007 to 2012) on a majority of the landscape in Iowa, though only about one quarter of new agriculture in Mato Grosso is correctly predicted. The proximity-based model performs as well in Iowa and slightly better in Mato Grosso (35% correct in pixel-specific changes). However, despite this similar performance, the agreement between new agricultural pixels for the two models is low (55% in Iowa and 31% in Mato Grosso). Thus, the pixels that are identified correctly by each model are different pixels, for the most part.

Supplementary Table 12 - Model performance

	Iowa	Mato Grosso
Logistic regression model	59%	27%
Proximity-based model	59%	35%

Despite this error in individual pixel-level conversion, the greater question to environmental impact assessment is whether the land use change model captures the types of land-use change trends that are important to ecosystem services. We therefore assess the difference between impacts on ecosystem services modeled from past agricultural expansion and LCM-generated agricultural expansion, and in this case find the magnitude of impacts to be relatively robust to pixel-level errors in land-change model prediction (see Figure 3, main manuscript).

To create a validation layer for the ecosystem service impacts predicted by the LCM model, we generate a binary map of where conversion to agriculture occurred between 2007 and 2012, and then overlay these pixels as new agriculture onto the 2007 landscape. The only impact for which we are unable to assess LCM uncertainty in this way is Water Consumption. Impacts resulting from past land-use change cannot be modelled because change in irrigation resulting from land-use change during this period is unknown. For the remaining impacts, predictions that show complete alignment with this map represent total land change model accuracy (assuming no error in the MODIS classifications). In this case our validation is only concerned with conversion from vegetated land to agriculture, and not any other categorical transitions. We then use the absolute amount of conversion between 2007 and 2012 as a new “demand scenario” to feed into the LUCI-LCA and calculate the impact per T of HDPE. This normalized impact can then be compared to the logistic regression model, as well as the proximity-based scenario generator (abbreviated as “SG:PB” for “Scenario Generator: Proximity Based”). SG:PB essentially creates a suitability layer as well, except this layer is derived purely based on distance to or from the frontier of certain land cover classes (in this case, agriculture). When running SG:PB, we also apply the same restrictions for the types of land that can be converted – specifically, omitting barren, urban, and water.

3.2. Modeling Biodiversity & Ecosystem Services Impacts from Agricultural Expansion and Intensification

Here we describe the methods, data, assumptions, and results from the ecosystem services modeling to assess impacts from the increased production to meet the different scenario demand targets. The spatially-explicit effects of agricultural expansion are modeled in Iowa and Mato Grosso, for carbon loss (InVEST Carbon Storage and Sequestration and Forest Carbon Edge Effects models), nitrogen export (InVEST Nutrient Delivery Ratio model), water consumption from irrigation (InVEST beta model for blue water consumption), sediment export (InVEST Sediment Delivery Ratio model), and biodiversity (MSA) reduction (InVEST GLOBIO model).

However, when it comes to modeling the impacts of intensification, our approach is only partial. We believe the science and data are not adequate to model the impacts of intensification to biodiversity, sediment export or carbon loss / sequestration, for two reasons: first, there are currently no globally available maps of crop-specific composition at the resolution needed for the InVEST models. It is therefore not possible to make predictions of the spatial effects of intensification of existing crops. Second, even if such data were available, we do not yet have a mechanistic model or even consistent scientific evidence linking yields to specific changes in tilling and soil management that may also affect erosion and carbon sequestration, or between yields and hospitability to biodiversity. We thus limit our analysis of impacts of intensification to nitrogen export and water consumption, via models predicting the relationship between yields, nutrient application, and irrigation.

In the following sections, we describe the individual InVEST models used to estimate ecosystem impacts to substitute key elements (inventory data and characterization factors) in the agricultural stage of standard LCA. The ecosystem impacts from the land use change scenarios, modeled using InVEST or simple GIS approaches, align to the LCA impact categories as follows:

1. Carbon Loss (input to Global Warming Potential)
2. Nutrient Export (input to Eutrophication Potential)
3. Water Consumption from Irrigation (input to Water Consumption and Global Warming Potential - energy used for pumping in irrigation)
4. Sediment Export (input to Erosion Potential)
5. MSA Reduction (input to Biodiversity Damage Potential)

3.2.1. Carbon Loss

3.2.1.1. Overview

We use the InVEST (v 3.2) carbon edge effects model to estimate loss of carbon storage for each scenario. The carbon edge effects model is an extension of the InVEST carbon model, which incorporates our recent work documenting the effects of fragmentation on carbon storage in tropical forest edges.[31] The model follows the typical inventory approach[32] for all habitat types other than tropical forest.

3.2.1.2. Inputs and Assumptions

The full description of the InVEST carbon edge model can be found in the InVEST User's Guide online.[33] For forest carbon edge, we included only estimates for below-ground, because above-ground carbon was predicted by the model based on a pixel's distance from forest edge. For consistency with the standard LCA approach (Ref. 13), wherever possible we use global estimates for carbon in different vegetation classes from IPCC (Ref. 32) and FAO Global Forest Resource Assessment[34] defined in the land use-land cover map we are using (MODIS, IGBP classification). Where coverage of certain classes is missing in those sources (e.g. savanna, shrubland), we use the dataset developed by Ruesch and Gibbs[35]. We summarize the input data sources and assumptions in Supplementary Table 13.

The effect of intensification practices, specifically normal and low tillage, on soil carbon storage are not well understood and this remains an open area of research. The majority of studies on this topic have only measured soil carbon within the first 20-30 cm, and meta-analyses[36,37] have shown that when lower depths of the soil profile are included, the effect of tilling is negligible. Furthermore, we have no quantitative relationship between tilling and yields; including the effects of management would only have been useful in terms of providing an upper and lower bound for the impacts on carbon storage. Therefore, we do not include the impacts of mechanization or intensification in our assessment of the effects of land use change on carbon storage. We do not report results for soil carbon here, because this parameter is already considered within the LCA and does not require substitution since we do not expect effects to be spatially explicit. Biophysical tables used in the model are reported in Supplementary Note 3.

Supplementary Table 13. Input data for the InVEST carbon model

Input	Source	Main assumptions
**LULC**	Land use land cover map<br>Source: MODIS, w/ logistic LCM	Cf. Section 3.1.2<br>Uncertainty tested by running alternative scenarios
**Carbon edge effects parameter table**	InVEST carbon model	See Ref. 31
**Biophysical table**	Ref. 35	Cross-walking GLC land cover to MODIS IGBP classification. See Supplementary Note 3

3.2.1.3. Model Sensitivity

We test parameter sensitivity for scenario 3 in the predominant land covers into which agriculture expanded in our land-change model for each region. In Mato Grosso, the predominant land cover transformed is woody

savanna (<80% of converted habitat for scenario 3; Supplementary Figure 4); in Iowa, it is forest (60%) and grassland (20%).

For Mato Grosso, the globally-available estimate for carbon stored in woody savanna is 53 T/ha (Table S1f in Ref. 35). We took upper and lower bound estimates from the literature for the Mato Grosso region, with carbon values ranging from 21.1. T/ha[38] to 71.9 T/ha.[39]

For Iowa, the carbon estimated in forest was 93 T/ha and in grassland it is 6.4 T/ha (both according to the Direct Land Use Change Assessment tool (Ref. 13) used in standard LCA). According to the literature, the upper bound for above and belowground carbon stored in forest in the Midwestern US is 159 T/ha[40] and the lower bound was 77 T/ha.[41] Carbon estimates for grassland in the region range from 11.9 T/ha[42] to 4.9 T/ha.[43]

3.2.2. Nutrient Export

3.2.2.1. Overview

We use the InVEST (v 3.2) nutrient model to estimate Nitrogen (N) export for each scenario. The full description of the InVEST nutrient model can be found in the InVEST User's Guide online.[44] For each pixel, the model computes the nitrogen load, i.e. the amount of nitrogen running off the pixel (either by surface or subsurface flow), and the transport coefficient, termed nutrient delivery ratio (NDR). NDR is a factor between 0 and 1 that represents the amount of nitrogen that actually reaches the stream, based on the landscape properties (slope, land cover, etc.) between the pixel and the stream.

3.2.2.2. Inputs and Assumptions

Generally, values for N loads and efficiencies are sourced from the InVEST parameter database (Supplementary Table 14). For the land-use change scenarios to meet the required production increase, N loads for new agriculture (sugarcane or maize) are computed as the product of fertilizer application rates and N use efficiency. Fertilizer application rates are based on ecoinvent 2.2 or local literature (Supplementary Table 14). N use efficiency is set to a global average value of 0.6[45]. Biophysical tables used in the model are reported in Supplementary Note 3.

Supplementary Table 14. Input data for the InVEST nutrient model

Input	Description and source	Main assumptions
**LULC**	Land use land cover map<br>Source: MODIS, w/ logistic LCM	Cf. Section 3.1.3<br>Uncertainty tested by running alternate scenarios
**DEM**	Digital Elevation Model (topography)<br>Source: SRTM	-
**N loads**<br>**N efficiencies**	Parameters describing the N inputs to the system (N loads), and the N retention (N efficiency) for each LULC<br>Source: InVEST database*, ecoInvent 2.2 and local studies**	For most LULC, parameter values are set to the average value for the state or the region<br>Loads for new agricultural land are specific to sugarcane or maize**<br>Loads for intensification areas are based on relative change in yield for each scenario
**Proportion subsurface**	Parameter describing the proportion of N load leaching to groundwater<br>Source: local studies**	Leaching only occurs on agricultural land
**Subsurface flow distance and efficiency**	Parameters describing the attenuation of N in groundwater flow<br>Source: Default values in InVEST	Default values are used since these parameters are difficult to find locally. Because of the low leaching rates, the model is not very sensitive to these parameters
**Threshold flow accumulation**	Iowa: 500<br>Mato Grosso: 600	-
**k,**	Default values	-

*This database was developed by the Natural Capital Project and comprises model parameter values sourced from hundreds of peer-reviewed studies. The database is available at:
http://naturalcapitalproject.org/pubs/BiophysicalParameter_database_7_Jun_2013.accdb

** For expansion sugarcane/maize: Mato Grosso: N application rate: 55 kg/ha (ecoinvent 2.2; Lopes, A.S., 2004. Fertilizer use by crop in Brazil. FAO, Rome), Leaching rate: 1.5% from Ref. 77; Iowa: N application rate: 157.2 kg/ha (ecoInvent 2.2; IFA, 2006. World Fertilizer Use Manual), leaching rate: 8% average value from Ref. 92 for the Corn Belt; N use efficiency: 0.6 (see text for details).

3.2.2.3. Impacts of Intensification

In reviewing the effect of intensification practices on nutrient dynamics (cf. Supplementary Note 4), we find impacts to be very heterogeneous. To represent intensification in Mato Grosso, we calculate the increase in fertilizer application necessary to reach the intensified yield (0), relative to baseline (average current) yields. While the availability of other nutrients (notably Phosphorus and Potassium) along with pesticides, seeds, machinery and knowledge can also limit the crop yields, we focus on N because N availability is considered the main limiting factor for crops in these regions.[46] For each production scenario, we then increase N loads for intensification areas by that percentage. (As noted in Section 3.1.1, no intensification is expected in Iowa.)

The additional N required to reach the intensification target is determined by reversing the underlying equations in the InVEST crop model, which are based on Mueller et al.'s[47] global study of fertilizer application rates and relationships to crop yields for 12 focal crops, including sugarcane. This relationship, a nonlinear regression, is derived independently for 100 different climate types or "bins", defined by growing degree days and annual precipitation. Climate bins are mapped at 10 x 10 km spatial resolution and the climate bin assigned to any spatial location is available in the InVEST crop model database.[48] To estimate the N application rate (N_{CB}, in kg/ha) required for sugarcane yield in a particular climate bin (CB), assuming no other nutrients are limiting, we apply the following equation (adapted from Equation S2 of the Supplemental Material in Ref. 47):

$$N_{CB} = \frac{-\ln \frac{1-(\frac{Y_{max_{CB}}}{Y_{mod}})}{b_N}}{c_N}$$

where $Y_{max_{CB}}$ is the maximum sugarcane yield attainable in a particular climate bin, Y_{mod} is the sugarcane yield for which the N application is being modeled, c_N and b_N are parameters defined by the nonlinear regression for sugarcane (Ref. 47). These variables are all available for each of the 12 staple crops in the InVEST crop model database.

This equation is applied to baseline yields as well as intensified yields under the different volume production scenarios in order to derive the N required to achieve the production level in each scenario (Supplementary Table 15). We take an area-weighted average of the N required to reach the yield target across the entire agricultural expansion area (which for simplicity is also where all intensification is assigned), based on the number of pixels in each climate bin (Supplementary Note 5). The application rates for the different scenarios are divided by the application rates for current yields to arrive at the percent increase in N application required for each scenario (Supplementary Table 15).

For example, in Mato Grosso, the baseline application rate for sugarcane in Brazil is 55 kg/ha (Ref. 46) and for scenario 3 the relative increase in fertilizer application to achieve the intensified yield is 28.1% (or increased by a factor of 1.281). With the N use efficiency of 0.6, this gives: load_n = 55 x 1.281 x (1-0.6) = 28.2 kg/ha.

Supplementary Table 15. Intensification modeled to reach yield target from current average sugarcane yields.

Scenario	Tonnes from intensification	Intensification Area	Increased N app rate for Intensified Yield
1	39840	7504	21.4%
2	157222	28059	27.3%
3	590469	104730	28.1%

3.2.2.4. Model Sensitivity and Verification

3.2.2.4.1. Sensitivity analyses

A major uncertainty in assessing nutrient impacts is related to N loads, i.e. sources of nutrients in the landscape. For agricultural land, these are driven by the fertilizer inputs and the amount of leaching. In the LUCI-LCA approach, we assess the effect of errors in this input by running the model for Scenario 3 with lower and upper bounds for these parameters.

Sugarcane in Mato Grosso

We use a weighted-average N application rate of 55 kg N/ha for sugarcane in Brazil based on an FAO report (Ref. 46). According to this study, the rates can vary from 14 kg N/ha in the North to 76 kg N/ha in the South. Other reports[49] suggests that N application rates are about 78 kg N/ha. In a following study[50], the author used an average of 60 kg N/ha, with the sugarcane yield of 72.52 T/ha. In a sensitivity analysis, he used minimum and maximum values of 35 kg N/ha and 97 kg N/ha, respectively, with a standard deviation of 16 kg N/ha. This range represents an error of approximately 45%, which we use in our analysis. Based on these uncertainty bounds, the additional N export from Scenario 3 changes by -42% and 37% relative to the original estimate.

Maize in Iowa

According to IFA 2006[51], the N application rate can range from 145 kg N/ha in the western part of the maize belt to 179 kg N/ha in the eastern part. This corresponds to a range of approximately 10%. In a US study by Grassini et al.[52], average N application rates ranged from 158 kg N/ha in Iowa, to 183 kg N/ha in Nebraska. Similar ranges are found in recommendations by Iowa State University[53] (140 to 190 kg N/ha), which leads us to consider a relative error of 15%. Based on these uncertainty bounds, the additional N export from Scenario 3 changed by -7% and 23% relative to the original estimate.

3.2.2.4.2. Model verification

To assess the credibility of our results, we compared InVEST nutrient export predictions with estimates from a global model (NEWS2) and from local empirical data (Supplementary Table 16). Comparison with NEWS2 and local sources suggest that InVEST underestimates total N export. This could be due the omission of other sources of nutrients in InVEST (e.g. point loads, especially in IA) and simplified representation of transport: in particular, the model simplifies the complex processes that drive nutrient degradation in surface and subsurface flows. However, the model correctly predicts Iowa as the location with the higher standardized N exports (i.e. per ha), which suggest that the relative difference between the scenarios is credible.

Supplementary Table 16. Comparison of InVEST results with alternative nutrient data, for total N export (kg/ha/yr).

	Sugarcane, Mato Grosso	Maize, Iowa
InVEST	0.26	1.66
NEWS2*	[1-2]	[2-3]
Other sources[54,55]	[4.1; 54]	[4-74]

*NEWS2 is a global model of nutrient emissions developed by Mayorga et al.[56] , which was calibrated against observations and accounts for both point sources and non-point sources of nutrients. See calculation details in the study by The Nature Conservancy available at: http://nature.ly/TNC-Dow-Brazil ("verification of hydrologic model predictions"). Here, we use the range for "pristine catchments" given the small proportion of agriculture in the baseline scenario.

3.2.3. Water Consumption

3.2.3.1. Methods

To assess the impact of agricultural expansion on water availability, we compute the irrigation water requirements and the resulting water consumption per tonne of product.

Net irrigation requirements, i.e. the amount of water needed by specific crops to grow, are computed based on a water balance at the monthly time scale[57], for each month of the growing period (April to August in Iowa, May to October in Mato Grosso). For each cropland pixel, and each month, the net irrigation requirements (Irr_n) are:

$$Irr_n = \begin{cases} k_c ET_0 - P_{avail} & if \quad k_c ET_0 > P_{avail} \\ 0 & otherwise \end{cases},$$

where k_c is the crop factor for the crop of interest, ET_0 is the reference evapotranspiration, and P_{avail} is the available precipitation, i.e. the amount of precipitation that did not leave as quick flow and is available to crops. P_{avail} is computed at the monthly time step with the InVEST index water model (cf. Supplementary Table 17, "Quickflow"), based on mean monthly precipitation and number of rain events. Given that the amount of quick flow was small (<5% relative to precipitation), with little variation between months and pixels, we set it to a constant value of 5%.

Net irrigation requirements are converted into predicted water consumption for irrigation. This is achieved by accounting for two factors: the irrigation efficiency, since some water extracted for irrigation is lost before reaching the crops; and the current irrigation rate, which is based on existing irrigation rates in the states of interest (some areas need irrigation in theory but are either restricted or lack equipment).

Irrigation efficiency is based on Rohwer et al. (Ref. 11; Supplementary Table 17), a dataset that provides more accurate information than the commonly used method developed by Doll and Siebert (Ref. 57). Note that irrigation efficiencies are averages for all crops, at the country level, in the absence of local data. The amount of water extracted to meet irrigation needs, or gross irrigation requirements (Irr_g), is:

$$Irr_g = \frac{Irr_n}{E}$$

Predicted irrigation volumes, i.e. the amount of water likely to be used by farmers in the field, are based on current irrigation rates. Due to lack of infrastructure, water regulations, or a decision not to irrigate, farmers do not always irrigate at the theoretical rates calculated above (Irr_g). We use current irrigation rates for all the expansion areas from global statistics: specifically, local data and a gridded dataset from Aquastat[58], which estimates the percentage of area actually irrigated as a function of areas equipped with irrigation (resolution of ~12km, see details in Supplementary Table 17). For each site, we compute the average percentage of area actually irrigated, AAI, and compute predicted irrigation volume (Irr_p) as:

$$Irr_p = Irr_g \times AAI$$

The water consumption (WC, in m^3/T product) only accounts for water evapotranspired or incorporated in a product and is therefore calculated as:

$$WC = \frac{Irr_p \times E}{Prod} = \frac{Irr_n * AAI}{Prod}$$

Where *Prod* is the amount of HDPE in tonnes.

3.2.3.2. Inputs and Assumptions

A summary of model inputs and assumptions is provided below.

Supplementary Table 17. Inputs to the blue water consumption model

	Description and source	Main assumptions/processing
Monthly precipitation	Gridded dataset at 30s resolution (~1km)<br>Source: WorldClim[14] [a]	Data from period 1950-2000
Monthly reference evapotranspiration	Gridded dataset at 30s resolution (~1km)<br>Source: CGIAR (original source is WorldClim data, cf. precipitation data[a])	Data from period 1950-2000

Kc	Crop factors for sugarcane or maize<br>Source: AquaStat[59] (Annex 2 provides Kc values per crop; Annex 4 provides irrigated crop calendar per country)	We assume crops are potentially irrigated (depending on needs) from planting month to harvest (April to August for maize, May to August for sugarcane)<br>For the baseline, given the lack of crop-specific information, we set Kc for all Ag to the value of the crop of interest (maize for Iowa, sugarcane for Mato Grosso). This is reasonable given that other crops will have similar crop coefficients, and the focus of the modeling is on the difference between baseline and expansion scenarios (so the error on other crops will cancel out).
Quickflow	InVEST Seasonal Water yield model (in development)	Quickflow is an intermediate output from the InVEST seasonal water yield model, simply based on the Curve Number approach (see InVEST seasonal water yield model[60]). For simplicity, we used a single soil type (hydrologic group B). Number of rainy days are obtained from local sources [b].
Irrigation efficiency	Ratio of water used by crops over water extracted for irrigation (Ref. 11)	
Areas actually irrigated	Gridded map of actual irrigation (based on equipment and decision to actually irrigate) for 2005<br>Source: FAO data (Siebert et al.[58])	Given the coarseness of the data, we average these data at the watershed scale to estimate the regional irrigation level.
Withdrawals	Total withdrawals per basin<br>Source: Aqueduct[61]	In the Aqueduct model, water is routed from one subwatershed to another, such that the blue water should be obtained from only the most downstream subwatershed (not summing blue water values across the watershed area). In IA, however, since some watershed areas belong to a different basin (HUC4 #10 vs #7 for most of IA), they are not draining to the same outlet). The blue water contribution from these subwatersheds is thus added to the main outlet (in basin #7) to obtain total blue water.
Available water	Total blue water per basin<br>Source: Aqueduct	

[a] MG data were all reprojected to Mercator since original projection (Brazil polyconic -SAD69)
[b] Number of rainy days: Iowa: http://www.usclimatedata.com/climate/iowa/united-states/3185; Mato Grosso: data.worldbank.org/developers/climate-data-api

3.2.3.3. Model Sensitivity and Verification

Regional assessments of irrigation water consumption comprise a number of uncertainties. We summarize and discuss the main sources of uncertainty in Supplementary Table 18 below. An important consideration is the time scale: in our calculations, hydrologic data are long-term averages to reduce the effect of climate variability, whereas some datasets represent recent years only (e.g., withdrawals). We do not assess the effect of these uncertainties individually.

Supplementary Table 18. Main sources of uncertainty for the quantitative estimates of water consumption

Source of uncertainty	Level of uncertainty
Precipitation inputs	Medium. Precipitation is from the 1950-2000 period to produce long-term results. Impacts of climate change are not considered in this analysis; they are likely to increase actual irrigation water consumption in Iowa
Reference evapotranspiration inputs	Low. Reference evapotranspiration, similar to precipitation, is computed for 1950-2000. See precipitation inputs for comments on climate change
Quick flow (runoff assumed to be unavailable to plants)	Low. Quick flow is estimated from InVEST based on monthly data (disaggregated to daily). Rerunning the IA assuming 0 quickflow (i.e. the extreme opposite), yields a difference in baseline requirements of 10%.
Error in crop coefficients*	Medium. Trials for IA yield a difference of 38% for June

Error in total withdrawals in total blue water (from Aqueduct)	Medium. Aqueduct data are based on 1950-2008. These estimates are from disaggregated country-level data using regression based on proxies for industrial, agricultural, and domestic uses. Data for global analysis may be uncertain.

*We note that model outputs are a linear function of these factors so the effect of their uncertainty can be assessed by propagating the error linearly.

3.2.3.3.1. Irrigation and water yield volumes

To verify predicted irrigation volumes in Iowa, we compare our estimates with USGS data.[62] In 2010, daily irrigation use in Iowa was 42.8 Mgal or 0.00016 km^3. Correcting for area since the watershed considered was larger than the state of Iowa, and multiplying over a year, this yields an annual irrigation water volume of 0.09 km^3. We note that this correction is approximate since areas in the North-east, outside the state of Iowa, are responsible for a large proportion of the irrigation requirements in this region. Similarly, we compare the total irrigated areas (116,000 ha vs. 115,000 ha, for USGS and our estimates, respectively), and the total withdrawals (6.5 km^3/yr vs. 9.7 km^3/yr, for USGS and our estimates, respectively). This verification suggests that the values for Iowa are reasonable first-order estimates.

In Mato Grosso, similar data are not available so we simply verify the amount of available blue water (from Aqueduct) with the uncalibrated InVEST annual water yield model (which uses the precipitation, reference evapotranspiration inputs). The error is 35% for Mato Grosso (and 16% for Iowa), indicating potential errors in model inputs but suggesting that estimates are credible.

3.2.3.3.2. Comparison with other studies' results

Although water consumption values (in m^3/T crop) are similar across scenarios, they are very sensitive to assumptions about actual water requirements. We compare our results to several other sources, as summarized in Supplementary Table 19. Our results differ from those of Mekonnen and Hoekstra (Ref. 10) in terms of magnitude and ranking (water consumption for sugarcane is higher than maize in our study, but lower according to Ref. 10). This could be due to the differences in data sources and processing (see 3.3.3), and points to large uncertainties in the calculations of water consumption metrics (Ref. 10).

Supplementary Table 19. Water consumption (in m^3/T crop) for sugarcane (Mato Grosso) and maize (Iowa). (Note, main results in manuscript are presented in m^3/T HDPE, but for direct comparison to other sources we keep units as water consumed per crop here.)

	Sugarcane (MG)	Maize (IA)
LUCI-LCA	2	1
LUCI-LCA Lower and Upper bounds*	[0.48; 2.5]	[0.18; 1.6]
Mekonnen and Hoekstra (Ref. 10)	0.7	2
Other sources	[0.7-1.85] [a]<br>0.3 [b]	~9 [c]

*For Mato Grosso: Lower bound is based on irrigation volumes needed for "salvage irrigation", described by Hernandes et al.[63] Upper bound is based on one standard deviation of the percentage of actually irrigated area across the region of interest (cf. Supplementary Table 18, we compute "Areas actually irrigated" from the average under the watershed).
For Iowa: Similar to the upper bound for Mato Grosso, the lower and upper bounds are based on one standard deviation of the percentage of actually irrigated area.
[a] Based on Ref. 63 [b] This number corresponds to the irrigation requirements assuming a "salvage irrigation" of 60 mm, for 12% of the sugarcane areas, based on data from Ref. 63 [c] Based on Shapouri et al.[64] This study suggests 5% irrigation in Iowa.

3.2.4. Sediment Export

3.2.4.1. Overview

We use the InVEST (v 3.2) sediment model to estimate sediment export for each scenario. The full description of the InVEST sediment model can be found in the InVEST User's Guide online.[65] The model computes the soil loss, i.e. the amount of sediment produced on each pixel, and the transport coefficient, termed sediment delivery ratio (SDR). SDR is a factor between 0 and 1 that represents the amount of soil loss that actually reaches the stream, based on the landscape properties (slope, LULC) between the pixel and the stream.

3.2.4.2. Inputs and Assumptions

To inform parameter selection, we review the effect of intensification practices on sediment dynamics (cf. Supplementary Note 4), finding the impacts to be very heterogeneous. Sediment export is more related to Best Management Practices than to the level of intensification. For this reason, we model all agricultural land with the same generic coefficients (the C and P factors of the sediment model, controlling soil loss in a pixel), thereby ignoring the distinction between standard and intensified production.

We summarize the input data sources and assumptions in Supplementary Table 20. Empirical values for the C (crop management) and P (practice) factors are derived from the InVEST database (see Supplementary Table 20 for details). When insufficient data is available for the state (either Mato Grosso or Iowa), we use regional data (South or North America). As noted above, C and P factors for both the baseline and expanded agricultural areas are set to the average of current agricultural land for the area (not specific to sugarcane or maize). Final biophysical tables used in the model are reported in Supplementary Note 3.

Supplementary Table 20. Input data for the InVEST sediment model

Input	Source	Main assumptions
**LULC**	Land use land cover map<br>Source: MODIS, w/ logistic LCM	Cf. Section 3.1.3<br>Uncertainty tested by running alternative scenarios
**Erosivity layer**	Iowa: USDA Isoerodent maps of the US;<br>Mato Grosso: Roose et al.[66] based on annual precipitation by WorldClim (Ref. 29)	
**Erodibility layer**	Iowa: USGS (STATSGO database)<br>Mato Grosso: SOTER database	
**DEM**	SRTM	-
**USLE C factor**<br>**USLE P factor**	InVEST database*	For each LULC, parameter values are set to the average value for the state or the region<br>Parameters for current and future agricultural land are assumed to be the same as general agriculture (not specific to sugarcane or maize)
**Threshold flow accumulation**	Iowa: 500<br>Mato Grosso: 600	-
**k, IC_0, SDR_{max}**	Default values	-

*This database was developed by the Natural Capital Project and comprises model parameter values sourced from hundreds of peer-reviewed studies. The database is available at: http://naturalcapitalproject.org/pubs/BiophysicalParameter_database_7_Jun_2013.accdb

3.2.4.3. Model Sensitivity and Verification

3.2.4.3.1. Sensitivity analyses

A major uncertainty in assessing sediment impacts is related to the C factors, i.e. which are empirical parameters representing the amount of soil loss relative to bare soil. As detailed in Supplementary Table 20, the LUCI-LCA approach uses regional parameters derived from the peer-reviewed literature. We assess the effect of errors in this input by running the model for Scenario 3 with an uncertainty bound of 50% around baseline values, which correspond to a typical error around these parameters (Hamel et al.[67], Chaplin Kramer et al.[68]). Based on these uncertainty bounds, the additional sediment export from Scenario 3 varies from -53% to 71%, and from -64% to 60%, respectively, in Iowa and Mato Grosso, relative to the original estimate.

3.2.4.3.2. Model verification

We verify the magnitude of InVEST predictions by comparing them with one global model (BQART[69]) and local empirical studies (Supplementary Table 21). Given the large uncertainties in sediment and nutrient modeling,

especially for ungauged basins,[70] it is outside the scope of this study to reduce modeling uncertainty in the sediment export estimates. The accuracy of these values impacts the results of the LUCI-LCA to the extent that absolute predictions are used. However, relative difference in sediment exports is more robust.

Supplementary Table 21. Comparison of InVEST results with alternative data for sediment export (T/ha/yr)

	Mato Grosso	Iowa
BQART	0.83	0.45
Regional studies*[54,55]	[0.04;0.74]	[0.2-17]
InVEST	0.71	2.1

* Garret (Ref. 54) for Iowa; Fonesca et al. (Ref. 55) for Mato Grosso. See calculation details in a study by The Nature Conservancy study at: http://nature.ly/TNC-Dow-Brazil ("verification of hydrologic model predictions")

Sediment yield in Mato Grosso is consistent with values from the BQART model (Supplementary Table 21). In Iowa, InVEST seems to overestimate the sediment yield; however, BQART is limited in the representation of agriculture (human modifications to a basin are represented by a factor that equals 1 for Mato Grosso, vs. 2 for Iowa), which certainly underestimates the impact of agriculture (97% of LULC). Overall, the order of magnitude predicted by InVEST is in line with BQART and regional studies.

3.2.5. MSA Reduction

3.2.5.1. Overview

We use the InVEST (v 3.2) GLOBIO model to estimate biodiversity impacts. GLOBIO uses a meta-analysis of studies around the world to derive changes in mean species abundance (MSA) resulting from different anthropogenic threats. We examine differences in MSA between baseline (2007) and scenario landscapes, and summarize the area-weighted average reduction in MSA and total affected area (through occupation or fragmentation) where biodiversity is impacted by land-use change

3.2.5.2. Inputs and Assumptions

The full description of the InVEST GLOBIO model can be found in the InVEST User's Guide online.[71] The three sources of threat considered in this model are land-use (affecting only the pixel of the new expanded agricultural land), fragmentation (affected by changes in nearby pixels), and infrastructure (affected by changes in nearby pixels; but not altered in the scenarios explored here). We summarize the data sources and assumptions in Supplementary Table 22.

Supplementary Table 22. Input data for the InVEST GLOBIO model

Input	Source	Main assumptions
**LULC**	Land use land cover map<br>Source: MODIS, w/ logistic LCM	Cf. Section 3.1.3<br>Uncertainty tested by running alternative scenarios
**Road map**	OpenStreetMap.org	Unnecessary to examine reductions in MSA from baseline; but included in order to assess differences in baseline between regions. More types of infrastructure could be included for a better estimate of total threat, but this is the best globally-available source.
**Pasture map**	Ramankutty et al.[72]	Yr 2000, 10 km resolution, proportional area in pasture
**Potential vegetation map**	Ramankutty & Foley[73]	50 km resolution, represents the world's vegetation cover that would most likely exist for 1986-1995 in equilibrium with present-day climate and natural disturbance, in the absence of human activities
**Primary threshold**	Set at 0.25 for both regions	Value for amount of primary habitat defined using FFQI to match GLOBIO database estimate in each region
**Pasture threshold**	Set at 0.5 for Iowa; 0.75 for Mato Grosso	Values for amount of pasture to match FAO estimates for each region

**Intensification threshold**	Set at 0.95 for Iowa; 0.5 for Mato Grosso	Values for proportion of intensification in each region, based on yield gap calculated by Foley et al.[74]
**MODIS to GLOBIO conversion table**	InVEST GLOBIO model	All forest types lumped; all grassland, savanna types lumped (partitioned by potential veg., pasture maps)
**MSA parameter table**	InVEST GLOBIO model	Based on meta-analysis in Alkemade et al.[75]; std. errors of mean effects included in uncertainty analysis

An MSA estimation ranges from 0 to 1, indicating the average proportional change in abundance of individual species in a location compared to the average abundance of the species within a pristine ecosystem. An MSA of 1.0 implies that, on average, species abundances are the same as in pristine land while an MSA of 0.0 implies that average species abundance is zero (i.e. locally extinct).

The typical use of MSA is to report an average value for a region, but when considering the impacts of localized agricultural expansion, relatively large local changes can be masked by the overall size of the landscape that is not changing. In our case study, the production scenarios convert <1% of the overall landscape. Biodiversity impacts should be considered in terms of their local rather than aggregate effects, in order to form a more conservative estimate of impacts especially for species whose ranges may be limited. We therefore subtract the scenario MSA maps from the baseline (2007) MSA maps for Iowa and Mato Grosso, and report results as averaged only over those pixels whose MSA values changed due to the agricultural expansion scenarios. This extends beyond the pixels that are actually converted from natural habitat to agriculture because the remaining habitat's configuration is altered by the conversion. Thus, MSA within unconverted habitat may still decline due to fragmentation resulting from agricultural expansion.

Because the method used in standard LCA multiplies the characterization factor in MSA by the area occupied or transformed (amortized for 20 years), we follow the same method to keep the two approaches as comparable as possible. However, we do not multiply only by the area of agricultural expansion, but the total impacted area, which includes both areas that have converted (from natural habitat to crop) as well as the areas that have been affected by fragmentation through their proximity to converted areas. (Supplementary Table 23).

Supplementary Table 23. Area of impact for Mean Species Abundance (MSA)

Study area	Crop	HDPE demand (T)	Area converted (ha)	Area impacted (ha)
MT	Sugarcane	23,000	7,504	12,850
MT	Sugarcane	86,000	28,059	56,875
MT	Sugarcane	321,000	104,730	209,075
IA	Maize	23,000	12,008	11,775
IA	Maize	86,000	44,898	42,825
IA	Maize	321,000	167,584	159,825

Impacted area could also be smaller than converted area if the area studied had very little habitat remaining, with some of that remaining habitat registering MSA values as low as the agriculture replacing it. In this case study, Mato Grosso has a larger area impacted than converted, as a result of fragmentation, while Iowa has a slightly smaller area impacted than converted

3.2.5.3. Model Sensitivity

To test the model's sensitivity to the error in MSA values for each land use related impact (land transformation, fragmentation, and infrastructure (e.g. roads)), we run the model with upper and lower bounds set at the mean MSA value plus or minus the standard error given in the meta-analysis by Alkemade et al. (Ref. 75). Based on these uncertainty bounds, the MSA by area impact from Scenario 3 varies from -51% to 68%, and from -32% to 11%, respectively, in Iowa and Mato Grosso, relative to the original estimate.

3.3. Integrating the predictive, spatially explicit information into LCA

The outputs of the individual InVEST models described in Section 3.2 are used to estimate ecosystem impacts and to directly substitute key elements (inventory data) in the agricultural stage of standard LCA. Adaptations, which transform the standard LCA into LUCI-LCA, are described next, for each of the LCA impact categories considered in this study.

3.3.1. Global Warming Potential

In LUCI-LCA, results of spatially explicit modelling substitute elements of the LCA, changing the estimates of carbon dioxide emissions from land use change. We also consider the spatially explicit impacts of agricultural intensification and irrigation on Global Warming Potential.

3.3.1.1. Greenhouse gas emissions from land use change

In LUCI-LCA, the output from the InVEST carbon edge effects model replaces results from the Direct Land Use Change Assessment tool (Ref. 13) that are used in the standard LCA. Specifically we replace the "CO_2 emissions from transformation" component of the life-cycle inventory (Figure 1, main manuscript).

The land use change (carbon loss) results provided from the InVEST model are based on the total amount of crop required to meet the demand for bio-HDPE for the different scenarios prior to allocation. In order to use these results in the LCA, they are allocated to HDPE, amortized over 20 years and converted into carbon dioxide equivalents (CO_2-eq.). The carbon dioxide emissions from land use change in the LCA are substituted with these updated results from the InVEST model.

The key points of difference between the approaches are; 1) emissions induced by land use change are spatially explicit in LUCI-LCA; 2) trends in land use change are evaluated on a regional (state) level (rather than country level) and; 3) impacts are based on the difference between current and predicted future change (rather than historical change over the last 20 years). The amount of different habitat types considered as changed are different between the two approaches, with standard LCA suggesting much more forest loss in Mato Grosso than is predicted by the logistic LCM, and no forest loss in Iowa, in contrast to LUCI (Supplementary Figure 4).

3.3.1.2. Greenhouse gas emissions from irrigation

Updated irrigation water volumes modelled as described in 3.2.3 are used to estimate the greenhouse gas emissions from the electricity required for pumping water during irrigation.

The sugarcane and maize datasets are updated to include irrigation based on 2.66% and 0.53% of the crop areas being irrigated respectively. The average volumes of water for irrigation of the sugarcane and maize used in the LUCI-LCA are calculated from irrigation volume (km^3) as given in Supplementary Table 24 and 25.

Supplementary Table 24. Irrigation volumes sugarcane (2.66% of crop irrigated)

Scenario	1	2	3
HDPE (T)	23,000	86,000	321,000
Irrigation water (km^3)	0.0032	0.0123	0.0463
Irrigation water (m^3T^{-1} sugarcane)	5.20	5.34	5.39
Consumed water (m^3T^{-1} sugarcane)	2.0	2.0	2.0

Supplementary Table 25. Irrigation volumes maize (0.53% of crop irrigated)

Scenario	1	2	3
HDPE (T)	23,000	86,000	321,000
Irrigation water (km^3)	0.00024	0.00088	0.00314
Irrigation water (m^3T^{-1} maize)	1.76	1.75	1.68
Consumed water (m^3T^{-1} maize)	1.0	1.0	0.9

3.3.1.3. Greenhouse gas emissions resulting from intensification (sugarcane)

Life cycle inventories for sugarcane cultivation are updated to consider the impact from intensification, although as noted previously maize is considered to be close to its maximum yield (See section 3.1). The intensification includes increase in yield and additional nitrogen fertilizer application.

3.3.1.3.1. Increase in yield

The percentage increase from the current spatially explicit weighted average yield to reach the theoretical intensified yield for each scenario is considered as given in Supplementary Table 26.

Supplementary Table 26. Calculating the increase in yield for each scenario

Scenario	Current yield (T/ha)	Theoretical intensified yield (T/ha)	Increase in yield
1	76.68	81.99	6.93%
2	76.39		7.33%
3	76.35		7.39%

In the LUCI-LCA, the yield increase is applied to the total results from the agricultural stage of the life cycle.

3.3.1.3.2. Increase in nitrogen fertilizer application

A factor representing the relative increase in N is derived from the nutrient model (section 3.2.2.3) and applied to derive the additional amount of N-fertilizers used (ammonium nitrate phosphate, ammonium sulphate, diammonium phosphate, potassium nitrate and urea) and therefore additional greenhouse gas emissions from their production is linearly derived. The increase in transport requirements to deliver the additional quantities of fertilizers to the farms is also considered, as well as the additional emissions of nitrous oxide, ammonia, nitrate and nitrogen oxides at the farm. Supplementary Table 27 provides a summary of inputs from InVEST used to estimate the intensification from an increase in nitrogen fertilizer application.

Supplementary Table 27. Inputs from InVEST used to estimate intensification from increase in nitrogen fertilizer application

Scenario	Tonnes from intensification	Increase in N
1	39,840	21%
2	157,222	27%
3	590,469	28%

An explanation of how to consider additional transportation is given in section 3.3.1.3.3 and N related emissions associated with additional fertilizer application are given in section 3.3.1.3.4. The greenhouse gas emissions induced are added to the Life Cycle Inventory, completing emissions occurring from the other agricultural activities.

3.3.1.3.3. Additional Transportation of N-Fertilizer

The additional transport steps required for the additional N-fertilizer are estimated using the following data from econinvent: 'RER: transport, freight, rail,' 'RER: transport, lorry >16t, fleet average' and 'RER: transport, lorry 3.5-16t, fleet average'. The contribution of the N-fertilizer transport steps in the 'BR: sugarcane, at farm' to other raw materials is based on the relative contribution of fertilizers given in tables 10.17 and 10.19 in Jungbluth et al. (Ref. 2), combined with the ratio of N to P_2O_5 and K_2O-fertilizers in the sugarcane dataset. The ratio of N to P_2O_5 and K_2O-fertilizers is based on weight of the fertilizers rather than the nutrient content (e.g. urea, as N refers to 1 kg N, which is 2.17 kg urea with an N-content of 46%). This results in the following life cycle inventory for the transport of nitrogen fertilizer per ha of sugarcane cultivation: RER: transport, freight,

rail [Railway]: 49.5 tkm, RER: transport, lorry >16t, fleet average [Flows]: 8.27 tkm and RER: transport, lorry 3.5-16t, fleet average [Street]: 1.32 tkm. The resource use and emissions from the transportation of additional N fertilizer are calculated by multiplication of these values by the fraction of relative N increase and attributing to the amount of product coming from intensification (Supplementary Table 15).

3.3.1.3.4. Additional N related emissions associated with fertilizer production and application

The factors applied to calculate emissions of ammonia, nitrous oxide and nitrate in sugar cane and maize inventories in ecoinvent assume a linear relationship between the levels of inputs per hectare and the level of emissions per hectare (Ref. 2; Nemecek et al.[76]). For consistency with the ecoinvent approach and IPCC approach (Ref. 32) the increase of N-related emissions per hectare are assumed to be directly proportional to the increase in N-fertilizer use per hectare. This means that the additional emissions from the application of additional fertilizer are calculated as a fraction of total emissions per ha, based on the factor describing the increase of fertilizer rate (Supplementary Table 27). These emissions are later attributed to the production volume, based on the estimated volume of crop that is coming from intensification (Supplementary Table 27).

3.3.2. Eutrophication Potential

The spatially explicit modelling of nutrient loss influences the values of nitrate leaching from the fields, which affects the Eutrophication Potential, along with intensification and irrigation.

Nitrogen export from the InVEST NDR model is substituted into standard LCA in place of the nitrate emissions to water in the agricultural inventories. The life cycle inventory for Brazilian sugarcane in ecoinvent 2.2 (Ref. 12) contains only a rough estimation of nitrate leaching, calculated with an emission factor of 2.5% of the N contained in the fertilizer, following work conducted by Stewart et al. for sugarcane fields in Australia.[77] Nitrate emissions for maize in ecoinvent 2.2 are based on the emission factor of 32% of the N contained in the fertilizer. This is based on field measurements from 1987 to 1994 according to Randall et al.[78]

Nitrogen loss results provided from the InVEST model are based on the total amount of crop required to meet the demand for bio-based HDPE for the different scenarios prior to allocation. In order to use these results in the LCA, they are allocated to the main product (HDPE).

There are several key points of difference between the standard and LUCI-LCA. In standard LCA, all the N that has the potential to leach to groundwater is assumed to reach the surface water. The LUCI-LCA approach considers the configuration of landscape and its effects on N leaching. Standard LCA inventories are based on single yield figures and N application rates, while LUCI-LCA uses spatially differentiated yield and N application relationships, based on climate. Additionally, there can be some inconsistency between data in some standard life cycle inventories depending on data availability (e.g., in this case, estimates for sugarcane are based on a modelling study, while those for maize are based on direct measurements of drained fields).

The updated impact for irrigation as given in section 3.3.1.2, and the additional impact from intensification, which includes increase in yield and increase in nitrogen fertilizer application as described in section 3.3.1.3 are added to the total result.

3.3.3. Water Consumption

The water consumption considered here is for irrigation only, and thus replaces the life-cycle inventory for the volume of consumed water during the irrigation process for crop production.

The main differences when compared to the LCA data, obtained from the WFN database[79], concern the data sources and processing. Irrigation requirements are calculated from different sources and processed differently; i.e. the water balance is calculated at the daily time step or the period 1996-2002 in LCA versus calculations at the monthly time step for 1950-2000, for LUCI-LCA. Land use maps are from different years (year 2000 from Monfreda et al.[80] for standard LCA; vs. 2007 MODIS data for LUCI-LCA, described in Section

3.1.3.2); and crop yields are obtained from two different studies (Reynolds et al.[81] in LCA vs. the yield data described in Supplementary Note 5 for LUCI-LCA). The difference in sources is necessary for the spatially-explicit modeling conducted in the LUCI-LCA approach.

The water consumption values for both standard and LUCI-LCA were also evaluated according to the AWARE impact assessment methodology to determine if the results changed when water scarcity of the two basins was included. In this case, it did not change the direction of the difference between the two feedstocks (see Supplementary Note 6 for more details).

3.3.4. Erosion Potential

In LUCI-LCA, the InVEST method for calculating sediment export (T/yr) is a direct replacement for the Saad et al. (Ref. 9) approach to calculating Erosion Potential within standard LCA for the agricultural phase of the life cycle. One of the main differences between the two approaches is the different reference state. The LUCI-LCA approach uses the current (in this case, 2007) state as a reference in contrast to the potential natural vegetation state used in standard LCA. The second main difference is the spatial resolution: whereas Saad et al.'s characterization factors for soil erosion are averaged across land use types and biomes for standard LCA, the LUCI-LCA approach describes a specific soil erosion for each pixel (here 500m). Third, as the standard LCA approach is based on the LANCA model,[82] it cannot differentiate between different agricultural practices, while LUCI-LCA has model parameters (the C factors from the USLE equation, cf. Section 3.2.4.2, Supplementary Note 3) that are specific to sugarcane and maize. Finally, and perhaps most importantly, standard LCA does not consider landscape configuration surrounding the occupied land. The LUCI-LCA approach models the retention of soil by the vegetation between the occupied land and the river, thus attenuating much of the potential soil eroded.

3.3.5. Biodiversity Damage Potential

In LUCI-LCA, the InVEST/GLOBIO method for calculating MSA impact is a direct replacement for the De Baan et al.[8] approach used to calculate Biodiversity Damage Potential within standard LCA for the agricultural stage of the life cycle. De Baan et al. also use MSA to estimate this biodiversity damage, but the method uses potential natural vegetation as the baseline from which to measure an effect. In LUCI-LCA we employ the current (2007) land use for the baseline. This is the greatest difference, and may in large part explain the orders of magnitude difference between the impacts estimated by the two methods. The difference between potential natural vegetation and currently occupied land is 0.84, many times larger than the average reduction in MSA on the impacted pixels found in both regions using the LUCI-LCA approach (~0.11-0.16).

The standard LCA method also adds the effects of transformation to those of occupation, both of which are compared to the same potential natural vegetation state (though transformation is amortized by 20 years to derive an annual figure, and occupation by the number of months out of the year the land is used for the crop in question). Thus, transformation impacts may be small compared to the occupation impacts for biodiversity damage when using the standard LCA method. In contrast, the LUCI-LCA method only considers transformation impacts of the future land use change predicted by the LCM (also amortized over 20 years, to follow convention). The difficulty with the interpretation of the standard LCA method for calculating biodiversity damage is the implication that occupation of land converted many years ago is linked to current impacts, when in reality the loss of biodiversity happened at the time of the change and cannot easily be reinstated. The greater current threat to biodiversity is the agricultural expansion, not the occupation of already converted land, hence our focus on land transformation only in LUCI-LCA.

Finally, the spatially explicit nature of the LUCI-LCA method allows several advances beyond the standard LCA approach. As previously mentioned, area impacted considers land that is reduced in quality due to fragmentation, not only transformation. Also, greater precision in current habitat types is possible due to the remotely sensed land cover data used, as compared to national or regional averages for standard LCA.

3.3.6. Sensitivity analysis

A sensitivity analysis is presented for scenario 3 (321,000 T HDPE) for the LUCI-LCA. Here we combined the analyses conducted for the standard LCA (section 2.4.) with those considered for the InVEST models described in sections: 3.2.1.3 for Carbon Loss, 3.2.2.4 for Nutrient Export, 3.2.3.3 for Water Consumption, 3.2.4.3 for Sediment Export, and 3.2.5.3 for MSA Reduction. There are, however, differences in terms of the data used for irrigation in the standard LCA (Supplementary Table 5) and the LUCI-LCA (Supplementary Table 28).

Supplementary Table 28. Consumed water (m^3/T crop) for sugarcane (Mato Grosso) and maize (Iowa) irrigation

	Base case	Lower	Upper
Sugarcane (LUCI-LCA)	2.05	0.48	2.54
Maize (LUCI-LCA)	0.91	0.17	1.54

The upper and lower bounds for total life cycle impacts (Global Warming Potential and Eutrophication Potential) and agricultural stage life cycle impacts (Water Consumption, Erosion Potential, Biodiversity Damage Potential) are shown in Supplementary Table 29 for scenario 3 of HDPE production (321,000 tonnes) in LUCI-LCA and standard LCA. Sensitivities for standard LCA are based on the assumption of yields (see Section 3.3.1.3.1), as described in Section 2.5. Sensitivities for LUCI-LCA are shown for InVEST parameter uncertainty only, as well as for total sensitivity to parameter uncertainty and yield uncertainty.

Supplementary Table 29. Sensitivity analysis for the effect of model parameters on estimated LCA impacts

	Global Warming Potential (T CO_2-eq /T HDPE)		Eutrophication Potential (T N-eq /T HDPE)		Water Consumption (m^3 water /T HDPE)		Erosion Potential (T sediment /T HDPE)		Biodiversity Damage Potential (MSA impact/T HDPE)	
	Maize	Sugarcane	Maize	Sugarcane	Maize	Sugarcane	Maize	Sugarcane	Maize	Sugarcane
LUCI-LCA lower bound (InVEST parameters)	9.9	5.2	0.013	0.001	1.0	12.9	1.1	0.2	0.0015	0.0036
LUCI-LCA upper bound (InVEST parameters)	13.6	5.3	0.015	0.002	9.0	67.9	4.1	0.9	0.0051	0.0059
LUCI-LCA lower bound (InVEST + yield sensitivity)	9.4	4.9	0.013	0.0009	0.9	11.7	1.0	0.2	0.0014	0.0033
LUCI-LCA upper bound (InVEST + yield sensitivity)	14.4	5.6	0.015	0.002	9.7	73.7	4.4	1.0	0.0056	0.0064
Standard LCA lower bound (yield sensitivity)	4.6	5.9	0.035	0.0007	1.6	1.8	10.6	8.0	0.25	0.66
Standard LCA upper bound (yield sensitivity)	4.7	6.7	0.038	0.001	17.5	11.5	12.7	9.6	0.37	0.97

Supplementary Note 1

Life Cycle Definitions

From the ILCD Handbook, General Guide for Life Cycle Assessment[83]:

"The attributional life cycle inventory modelling principle is also referred to as 'accounting', 'book-keeping', 'retrospective', or 'descriptive' (or sometimes and potentially confusing: 'average' or 'non-marginal'). It depicts the potential environmental impacts that can be attributed to a system (e.g. a product) over its life cycle. In attributional modelling, the system is hence modelled as it is or was (or is forecasted to be)."

"The consequential life cycle inventory modelling principle is also called 'change-oriented', 'effect-oriented', 'decision-based', 'market-based' and (older and incompletely / misleadingly capturing the issue: 'marginal' or 'prospective'). It aims at identifying the consequences that a decision in the foreground system has for other processes and systems of the economy, both in the analysed system's background system and on other systems. It models the analysed system around these consequences. The consequential life cycle model is hence not reflecting the actual (or forecasted) specific or average supply-chain, but a hypothetic generic supply-chain is modelled that is prognostizised along market mechanisms, and potentially including political interactions and consumer behaviour changes."

Supplementary Note 2

Land-Use Change Trends in Iowa and Mato Grosso

Land Use in Iowa, 1997-2006 (percent of total area of state by land use)

Iowa: 14,574,381 ha	1997	2002	2007	2012
Woodland and pastured woodland	3.91	3.71	3.31	3.24
Pasture and pastured cropland	9.97	8.58	7.62	5.92
Cropland (excluding pastured cropland)	71.21	71.63	70.77	72.28

Source: USDA-NASS[84]

Land Use in Mato Grosso, Brazil 1970-2006 (percent of total area of state)

Mato Grosso: 90,335,677 ha	1970 *	1975	1980	1985	1996	2006
Forest	21.05	37.63	44.95	43.22	46.25	39.05
perennial crops	0.15	0.22	0.44	0.42	0.35	0.84
annual crops	1.69	2.43	4.78	6.10	7.06	12.68
pasture land	77.08	59.59	49.66	50.19	46.20	47.32
planted forest	0.04	0.12	0.17	0.08	0.15	0.12
% total farm area/State area	0.19	0.24	0.38	0.42	0.55	0.53

Source: Soler et al. 2014[85]

Supplementary Note 3

Biophysical tables used in InVEST models

Carbon

lucode	LULC_desc	Total C (IA)	Total C (MT)*	C_below (MT)*	Source (IA)	Source (MT)
0	Water	0	0	0		
1	Evergreen Needleleaf forest	93	n/a	52.2	EC(2010)[86]	CDIAC[87]-Table S1b
2	Evergreen Broadleaf forest	93	n/a	52.2	EC(2010)	CDIAC -Table S1a
3	Deciduous Needleleaf forest	93	n/a	24.8	EC(2010)	CDIAC -Table S1b
4	Deciduous Broadleaf forest	93	n/a	24.8	EC(2010)	CDIAC -Table S1a
5	Mixed forest	93	n/a	38.5	EC(2010)	CDIAC – Table S1ab
6	Closed shrublands	7.4	53	0	CDIAC -Table S1f	CDIAC -Table S1f
7	Open shrublands	7.4	53	0	CDIAC -Table S1f	CDIAC -Table S1f
8	Woody savannas	14.2	53	0	Qui & Turner[88]	CDIAC -Table S1f
9	Savannas	14.2	53	0	Qui & Turner	CDIAC -Table S1f
10	Grasslands	6.4	7.6	0	IPCC[89]	IPCC
11	Permanent wetlands	10	0	0	Qui & Turner	n/a
12	Croplands	0	0	0	n/a	n/a
13	Urban and built-up	0	0	0	n/a	n/a
14	Cropland/Natural mosaic	5	0	0	CDIAC-Table S1i	n/a
16	Barren or sparsely vegetated	0	0	0	n/a	n/a
17	Maize/Sugarcane expansion	0	n/a	0	n/a	n/a

*Note: all values listed in Total C are carbon stocks for above- and below-ground combined, for all land covers except forest in Mato Grosso, for which below is listed separately, to be added to above-ground estimates generated by edge effects model.

Water models (sediment, nutrient, water availability)

Iowa

LULC code	LULC_desc	% LULC	usle_c	usle_p	load_n	eff_n	crit_length	prop_sub_N	Kc	root_depth	LULC_veg
0	Water	0%	0.0001	1	0	0.5	10	0	1.05	1	0
1	Evergreen Needleleaf forest	0%	0.03	1	2.2	0.9	300	0	1	3000	1
2	Evergreen Broadleaf forest	0%	0.03	1	2.2	0.9	300	0	1	3000	1
3	Deciduous Needleleaf forest	0%	0.03	1	2.2	0.9	300	0	1	3000	1
4	Deciduous Broadleaf forest	0%	0.03	1	2.2	0.9	300	0	1	3000	1
5	Mixed forest	0%	0.03	1	2.2	0.9	300	0	1	3000	1
6	Closed shrublands	0%	0.04	1	3.5	0.6	300	0	0.8	2150	1
7	Open shrublands	0%	0.1	1	3.5	0.6	300	0	0.5	2150	1
8	Woody savannas	0%	0.03	1	1	0.5	150	0	0.9	1070	1
9	Savannas	0%	0.03	1	1	0.5	150	0	0.9	1070	1
10	Grasslands	0%	0.03	1	1	0.5	150	0	0.9	1070	1

11	Permanent wetlands	0%	0.001	1	1.6	0.5	30	0	1.1	1	0
12	Croplands	78%	0.24	0.9	7.7	0.5	25	0.08	0.7 1	500	0
13	Urban and built-up	1%	0.1	0.5	6	0.1	10	0	0.4	1	0
14	Cropland/Natural vegetation mosaic	19%	0.135	0.95	4.35	0.5	150	0	0.8 6	1750	1
16	Barren or sparsely vegetated	0%	0.9	1	6	0.1	10	0	0.7	1075	1
17	Maize-expansion		0.24	0.9	76	0.5	25	0.08		0.74	1

Mato Grosso

lucode	LULC_desc	%LULC	usle_c	usle_p	load_n	eff_n	crit_length	prop_sub_N	Kc	root_depth	LULC_veg
0	Water	0%	0.0001	1	0	0.5	10	0	1.05	1	0
1	Evergreen Needleleaf forest	0%	0.005	1	4.2	0.8	300	0	1	3000	1
2	Evergreen Broadleaf forest	54%	0.005	1	4.2	0.8	300	0	1	3000	1
3	Deciduous Needleleaf forest	0%	0.005	1	4.2	0.8	300	0	1	3000	1
4	Deciduous Broadleaf forest	0%	0.005	1	4.2	0.8	300	0	1	3000	1
5	Mixed forest	0%	0.005	1	4.2	0.8	300	0	1	3000	1
6	Closed shrublands	0%	0.04	1	3.5	0.6	300	0	0.8	2150	1
7	Open shrublands	0%	0.1	1	3.5	0.6	300	0	0.5	2150	1
8	Woody savannas	1%	0.015	1	1.5	0.75	150	0	0.9	1070	1
9	Savannas	27%	0.025	1	1.5	0.75	150	0	0.9	1070	1
10	Grasslands	2%	0.02	1	1.5	0.75	150	0	0.9	1070	1
11	Permanent wetlands	0%	0.001	1	1.6	0.5	30	0	1.1	1	0
12	Croplands	9%	0.17	0.8	10	0.5	25	0.01	0.79	500	0
13	Urban and built-up	0%	0.1	0.5	6	0.1	10	0	0.4	1	0
14	Cropland/Natural vegetation mosaic	6%	0.0975	0.9	5.75	0.625	150	0	0.9	1750	1
16	Barren or sparsely vegetated	0%	0.9	1	6	0.1	10	0	0.7	1075	1
17	Sugarcane-expansion		0.17	0.8	*	0.5	25	0.01	0.9	1	0

***** Specific to each production scenario. For Yield A: (1) 26.72; (2) 28.01; (3) 28.18; (4) 28.12; (5) 27.91.

Supplementary Note 4

Effect of intensification practices on ES and their modeling

Note: the assumptions summarized below synthesize the information in the references and indicates the value selected for the InVEST model.

Iowa

Intensification practice	Effect on sediment retention modeling	Effect on nutrient retention modeling	Effect on water supply modeling
Conventional tillage	**C values decrease**<br>C decreases by 90%[90]<br>Empirical evidence[91]<br>*Assumption*: C is reduced by 90% wrt to conventional	**Increase in leaching**<br>Very low significance[92]<br>**Reduction in retention efficiency**<br>Empirical evidence[92]<br>*Assumption*: no change in leaching rate<br>Retention efficiency is reduced by 20% based on Table S3 and effect of no-till in the Meta-model	-
Fertilizer increase	-	**Increase in load**<br>Information on fertilizer management suggest that a decrease by 10% is a conservative assumption with precision agriculture[93]<br>*Assumption*: load is reduced by 90% wrt the "conventional practice" value.	-
Irrigation	-	**Increase in leaching**<br>Empirical evidence[91,92]<br>*Assumption*: leaching (proportion of subsurface flow) is reduced by ~90% based on Table S3 and effect of irrigation in the Metamodel	**Decrease in water recharge**<br>Additional irrigation may help increase yields. Amount is proportional to the plant water deficit (difference between precipitation and potential evapotranspiration)

Mato Grosso

Intensification practice	Effect on sediment retention modeling	Effect on nutrient retention modeling	Effect on water supply modeling
Conventional tillage	**C values increase**<br>Measured change of 90%[94]<br>*Assumption*: C is reduced by 90% wrt to conventional (NB: this is consistent with assumption for IA)	**Increase in leaching**<br>Empirical evidence[95]<br>*Assumption*: effect lumped with that of irrigation	-
Fertilizer increase	-	**Increase in load**<br>Empirical evidence[95,96]<br>**Increase in leaching**<br>Empirical evidence [95,96]<br>**Reduction in retention efficiency**<br>*Assumption*: Efficiency increased by 50% based on measurements[97]<br>*Assumption*: reduced load by 10% based on insights from precision agriculture[97]	-
Irrigation	-	**Increase in leaching**<br>Empirical evidence[91,95,96]<br>*Assumption*: reduced leaching by 50% based on measurements[97]	**Increase in water scarcity**<br>Water balance approach to increase yields.

Supplementary Note 5

Parameter table for crop yield-N application model.

Climate bins included in this table are only those represented in the agricultural regions of Mato Grosso (no intensification is expected in Iowa; see Section 3.1.1). The climate-bin intensification approach is based on the paper by Mueller et al. (Ref. 47), developed by the Global Landscapes Initiative at University of Minnesota's Institute on the Environment. For full parameter table with all global climate bins for sugarcane and other crops, download the datasets supporting the InVEST crop model at:

http://data.naturalcapitalproject.org/invest_crop_production

CB	Proportion of pixels								
	Sc1	Sc2	Sc3	c_N	b_N	Ymax	Current N per ha	Yield (A) N per ha	Yield (B) N per ha
20	0.00	0.00	0.00	0.0203	0.9513	95.27	74.4171424	94.4069139	87.5329307
37	0.00	0.00	0.00	0.0171	0.6654	104.8	50.3458598	65.3290574	60.4317608
38	0.00	0.00	0.00	0.0162	0.685	104.8	55.0808604	70.9371355	65.7544712
39	0.23	0.18	0.11	0.0195	0.8867	96.84	71.1536169	90.2085256	83.7344925
40	0.35	0.25	0.17	0.0165	0.9575	109.37	68.0896091	81.2884075	77.0307323
46	0.00	0.00	0.00	0.0246	0.8514	87.36	74.1210011	106.960703	94.1124561
47	0.00	0.00	0.00	0.0175	0.8742	103.18	67.0380093	82.6377456	77.5090256
48	0.02	0.04	0.06	0.0188	0.8642	102.92	62.3876742	77.1201131	72.2716775
49	0.06	0.06	0.06	0.024	0.8915	96.84	57.940528	73.3965734	68.1452782
50	0.05	0.06	0.08	0.0178	0.8819	103.18	66.453598	81.8020787	76.7559639
56	0.00	0.00	0.00	0.0162	0.7033	102.92	59.4431674	76.4789401	70.8724789
57	0.00	0.00	0.00	0.0161	0.6591	96.84	67.6308956	90.6809017	82.8495074
58	0.00	0.00	0.01	0.0325	0.8393	86.98	56.4200878	82.5038065	72.1682821
59	0.00	0.01	0.02	0.0249	0.8557	96.84	54.1301476	69.0086694	63.9535917
60	0.03	0.05	0.11	0.0176	0.9015	102.92	68.7505487	84.4199447	79.2631571
69	0.07	0.05	0.06	0.0246	0.8557	96.84	54.8745527	69.957686	64.8330902
70	0.19	0.31	0.33	0.0146	0.8953	96.84	95.3547058	120.713024	112.097365
78	0.00	0.00	0.00	0.025	0.8393	86.98	73.3418302	107.248684	93.813287
85	0.00	0.00	0.00	0.0077	0.9058	114.17	127.151471	151.584497	143.782807
88	0.00	0.00	0.00	0.0134	0.7146	86.98	125.249827	188.718649	163.569491
89	0.00	0.00	0.00	0.0128	0.5365	86.98	108.609372	175.013943	148.701512
98	0.00	0.00	0.00	0.0104	0.7437	96.84	115.99989	151.587223	139.496188
99	0.00	0.00	0.00	0.0108	0.6847	96.84	104.465839	138.867588	127.179363

Supplementary Note 6

Comparison of Water Consumption Results Using AWARE Methodology

The AWARE characterisation factors are given with two spatial resolutions (watershed and country) and two temporal resolutions (monthly and annual). We use the watershed values aligned with the spatially-explicit predicted prediction of agricultural expansion from our LCM. We use the annual agricultural average because we do not know the exact months of irrigation. The annual agricultural average is an average of the monthly values based on agricultural water consumption usually happening in this watershed, so it provides a general picture of the region (assuming the crop of interest is not being irrigated at completely different times compared to other crops).

The water scarcity footprint is calculated by multiplying the water consumption (inventory) in m^3 by the AWARE characterisation factor of the specified time and place (in m^3-eq / m^3)called and is expressed in m^3-eq. The methodology is based on The WULCA consensus paper submitted by Boulay et al.[98] The AWARE methodology and data are available at: http://wulca-waterlca.org/project.html.

Iowa

There are two watersheds in Iowa in the AWARE dataset. The watershed that covers most of the state and intersects with the agricultural expansion in our model has an agricultural annual average of 1.2. Therefore the water consumption in all Iowa scenarios (in m^3) is multiplied by this factor 1.2 to obtain results in m^3-eq.

Mato Grosso

There are three watersheds in Mato Grosso in the AWARE dataset. One watershed that accounts for 75% of the agricultural expansion has an agricultural annual average of 0.5, while another watershed that accounts for the remaining 25% of the agricultural expansion has a value of 1.1. Weighting the average by area produces a characterization factor of 0.65. Therefore the water consumption in all Mato Grosso scenarios (in m^3) is multiplied by this factor 0.65 to obtain results in m^3-eq.

Water scarcity footprint results (Scenario 3 volumes):

	Standard LCA		LUCI -LCA	
	Maize	Sugarcane	Maize	Sugarcane
Water Consumption (m^3 / T HDPE)	9.5	8.5	5.3	54.8
AWARE Characterisation Factor	1.2	0.65	1.2	0.65
Water Scarcity Footprint	11.4	5.5	6.4	3635.6

While the characterization factor suggests higher impact per unit of water used in Iowa than in Mato Grosso, this weighting is not enough to change the overall LUCI-LCA result (i.e., Mato Grosso has a higher water scarcity footprint than Iowa, for the case study scenarios). In LUCI-LCA, use of AWARE moderates the results such that sugarcane goes from being 10 times worse than maize to being ~ 6 times worse. In standard LCA, however, use of AWARE results in the water scarcity impact of maize changing from being roughly on par with sugarcane to being nearly twice as impactful.

Supplementary References

[1] Chaplin-Kramer, R., R. P. Sharp, L. Mandle, S. Sim, J. Johnson, I. Butnar, L. Milà i Canals, B. A. Eichelberger, I. Ramler, C. Mueller, N. McLachlan, A. Yousefi, H. King, and P. M. Kareiva. 2015. Spatial patterns of agricultural expansion determine impacts on biodiversity and carbon storage. Proceedings of the National Academy of Sciences 112:201406485.

[2] Jungbluth, N.; Chudacoff, M.; Dauriat, A.; Dinkel, F.; Doka, G.; Emmenegger, M. F.; Gnansounou, E.; Kljun, N.; Spielmann, M. Life Cycle Inventories of Bioenergy; Final report ecoinvent v2.0 No. 17; Swiss Centre for Life Cycle Inventories, 2007.

[3] Kochar, N. K., Merims, R., & Padia, A. S. (1981). Ethylene from ethanol. Chemical Engineering Progress, 77(6), 66–70 Jungbluth

[4] Tsiropoulos, I.; Faaij, A. P. C.; Lundquist, L.; Schenker, U.; Briois, J. F.; Patel, M. K. 2015. Life cycle impact assessment of bio-based plastics from sugarcane ethanol. J. Clean. Prod. 2015, 90, 114-127

[5] IPCC, 2013: Climate Change 2013: The Physical Science Basis. Contribution of Working Group I to the Fifth Assessment Report of the Intergovernmental Panel on Climate Change [Stocker, T.F., D. Qin, G.-K. Plattner, M. Tignor, S.K. Allen, J. Boschung, A. Nauels, Y. Xia, V. Bex and P.M. Midgley (eds.)]. Cambridge University Press, Cambridge, United Kingdom and New York, NY, USA, 1535 pp, doi:10.1017/CBO9781107415324.

[6] Goedkoop M.J., Heijungs R, Huijbregts M., De Schryver A.;Struijs J., Van Zelm R, ReCiPe 2008, A Life Cycle Impact Assessment Method Which Comprises Harmonised Category Indicators at the Midpoint and the Endpoint Level (version 1.08). Report I: Characterisation (first ed.) (2013) Available online at: http://www.lcia-recipe.net/file-cabinet (accessed 16.12.15)

[7] Globio (2015) http://www.globio.info/what-is-globio/reference-database/per-pressure

[8] de Baan, L.; Alkemade, R.; Koellner, T., 2013: Land use impacts on biodiversity in LCA: a global approach, The International Journal of Life Cycle Assessment, 18, 1216-1230

[9] Saad R, Koellner T, Margni M (2013): Land use impacts on freshwater regulation, erosion regulation, and water purification; a spatial approach for global scale level. Int J Life Cycle Assess 18: 1253-1264

[10] Mekonnen, M. M., & Hoekstra, A. Y. (2010). The green, blue and grey water footprint of crops and derived crop products, Value of Water Research Report Series No. 47. Delft, the Netherlands.

[11] Rohwer, J., Gerten, D., Lucht, W., 2007. Development of functional irrigation types for improved global crop modeling. Potsdam

[12] Nemecek, T., & Kägi, T. (2007). Life cycle inventories of agricultural production systems. (A. R.-T. R. S. ART, S. C. for L. C. Inventories, Zurich, & D. CH, Eds.). Retrieved from www.ecoinvent.ch

[13] BlonkConsultants. (2014). Direct Land Use Change Assessment Tool. Version 2014.1. Gouda. Retrieved from http://blonkconsultants.nl/en/tools/land-use-change-tool.html

[14] BSI, P. A. S. (2012). 2050-1: 2012 Assessment of Life Cycle Greenhouse Gas Emissions from Horticultural Products—Supplementary Requirements for the Cradle to Gate Stages of GHG Assessments of Horticultural Products Undertaken in Accordance with PAS 2050. British Standards Institution, London, UK.

[15] World Business Council for Sustainable Development, World Resources Institute, 2001. The greenhouse gas protocol: a corporate accounting and reporting standard. World Resources Inst

[16] FoodSCP, 2013. Envifood Protocol. Environmental Assessment of Food and Drink Protocol

[17] Zah R. and Hischier R. (2007) Life Cycle Inventories of Detergents. Final report ecoinvent data v2.0, No. 12. Swiss Centre for Life Cycle Inventories, Dübendorf, CH.

[18] Frischknecht R., Tuchschmid M., Faist Emmenegger M., Bauer C. and Dones R. (2007) Strommix und Stromnetz. In: Sachbilanzen von Energiesystemen: Grundlagen für den ökologischen Vergleich von Energiesystemen und den Einbezug von Energiesystemen in Ökobilanzen für die Schweiz (ed. Dones R.). ecoinvent report No. 6, v2.0. Swiss Centre for Life Cycle Inventories, Dübendorf, CH.

[19] Faist Emmenegger M., Heck T. and Jungbluth N. (2007) Erdgas. In: Sachbilanzen von Energiesystemen: Grundlagen für den ökologischen Vergleich von Energiesystemen und den Einbezug von Energiesystemen in Ökobilanzen für die Schweiz (ed. Dones R.). Swiss Centre for Life Cycle Inventories, Dübendorf, CH.

[20] European Commission (2007) Reference Document on Best Available Techniques in the Production of Polymers. Table 3.11. Page 64. August 2007 http://eippcb.jrc.ec.europa.eu/reference/BREF/pol_bref_0807.pdf.

[21] Sea-Distances.org. (2015). Sea Distances / Port Distances - online tool for calculation of distances between seaports. Retrieved from www.sea-distances.org

[22] Spielmann, M., Bauer, C., Dones, R., Tuchschmid, M., 2007. Transport Services, ecoinvent report No. 14. Swiss Centre for Life Cycle Inventories, Dübendorf, Switzerland.

[23] Brazil: Food and Agriculture Organization of the United Nations. (2012). FAOSTAT Database. Rome, Italy: FAO. Retrieved March 18, 2015 from http://faostat3.fao.org/download/Q/QC/E
Iowa: U.S. Department of Agriculture National Agricultural Statistics Service, 2015. NASS QuickStats Ad-hoc Query Tool. Retrieved March 18, 2015 from http://quickstats.nass.usda.gov/results/218D55ED-9057-3535-8C6E-6AB04DDE5383
[24] EarthStat. Yield gaps and climate bins for major crops. http://www.earthstat.org/data-download/
[25] The spatial location of the area to be allocated is determined after the breakdown between intensification and expansion has been determined, but there is no interaction between suitability as predicted in the logistic regression and yield.
[26] M. A. Friedl et al., MODIS Collection 5 global land cover: Algorithm refinements and characterization of new datasets. Remote Sens. Environ. 114, 168–182 (2010).
[27] While linear regression is sometimes applied directly to 0/1 outcomes, such an approach allows for violation of desired features, such as constraining output to be between zero and one.
[28] http://sedac.ciesin.columbia.edu/data/set/groads-global-roads-open-access-v1
[29] Hijmans, R.J., Cameron, S.E., Parra, J.L., Jones, P.G., Jarvis, A., 2005. Very high resolution interpolated climate surfaces for global land areas. Int. J. Climatol. 25, 1965–1978.
[30] 2001 and 2012 were the first and last years of MODIS data available at the time of model generation, and 2007 was chosen as a midpoint to provide adequate variation between the two
[31] Chaplin-Kramer, R., I. Ramler, R. Sharp, N. M. Haddad, J. S. Gerber, P. C. West, L. Mandle, P. Engstrom, A. Baccini, S. Sim, C. Mueller, and H. King. 2015. Degradation in carbon stocks near tropical forest edges. Nature Communications 6:10158.
[32] Intergovernmental Panel on Climate Change (IPCC). 2006. IPCC Guidelines for National Greenhouse Gas Inventories. Volume 4: Agriculture, Forestry and Other Land Use.
[33] Sharp et al. 2015. InVEST 3.2.0 User's Guide for the Forest Carbon Edge Effect model. The Natural Capital Project. Available at: http://data.naturalcapitalproject.org/nightly-build/invest-users-guide/html/carbon_edge.html
[34] http://www.fao.org/forestry/fra/fra2010/en/
[35] Ruesch, A., and H. Gibbs. 2008. New IPCC Tier-1 Global Biomass Carbon Map For the Year Laboratory, 2000. Carbon Dioxide Information Analysis Center (Oak Ridge National Laboratory, Oak Ridge, TN, USA). Available online at: http://cdiac.ornl.gov/epubs/ndp/global_carbon/carbon_documentation.html
[36] Blanco-Canqui, H., and R. Lal. 2008. No-tillage and soil-profile carbon sequestration: An on-farm assessment. Soil Science Society of America Journal 72:693–701.
[37] Baker, J. M., T. E. Ochsner, R. T. Venterea, and T. J. Griffis. 2007. Tillage and soil carbon sequestration—What do we really know? Agriculture, Ecosystems & Environment 118:1–5.
[38] Grace, J., J. San José, P. Meir, H.S. Miranda, R.A. Montes. 2006. Productivity and carbon fluxes of tropical savannas Journal of Biogeography 33(3):387 - 400. DOI:10.1111/j.1365-2699.2005.01448.x
[39] Fearnside P.M., et al. (2009) Biomass and greenhouse-gas emissions form land-use change in Brazil's Amazonian "arc of deforestation": The states of Mato Grosso and Rondônia. For Ecol Manage 258:1968–1978
[40] Smith JE, Heath LS, Skog KE, Birdsey RA (2006) Methods for calculating forest ecosystem and harvested carbon with standard estimates for forest types of the United States. United States Dept. of Agriculture, Forest Service, Northeastern Research Station. http://www.nrs.fs.fed.us/pubs/gtr/ne_gtr343.pdf
[41] Qiu, J., & Turner, M. G. (2013). Spatial interactions among ecosystem services in an urbanizing agricultural watershed. Proceedings of the National Academy of Sciences, 110(29), 12149-12154
[42] Kovacs, K., Polasky, S., Nelson, E., Keeler, B. L., Pennington, D., Plantinga, A. J., et al. (2013). Evaluating the return in ecosystem services from investment in public land acquisitions. Plos One, 8(6), e62202. Retrieved from http://dx.doi.org/10.1371%2Fjournal.pone.0062202
[43] Polasky, S., Nelson, E., Pennington, D., & Johnson, K. (2011). The impact of land-use change on ecosystem services, biodiversity and returns to landowners: A case study in the state of minnesota. Environmental and Resource Economics, 48(2), 219-242. doi:10.1007/s10640-010-9407-0
[44] Sharp et al. 2015. InVEST 3.2.0 User's Guide for the Nutrient Delivery Ratio model. The Natural Capital Project. Available at: http://data.naturalcapitalproject.org/nightly-build/invest-users-guide/html/ndr.html
[45] Brentrup, F., Palliere, C., 2010. Nitrogen use efficiency as an agro-environmental indicator, In: Proceedings of the OECD Workshop on Agrienvironmental Indicators, March 23-26. Leysin, Switzerland. Available at: www.oecd.org/tad/sustainable-agriculture/44810433.pdf
[46] FAO. Fertilizer use by crop in Brazil. Land and Plant Nutrition Management Service, Land and Water Development Division, Rome. Available at: http://www.fao.org/3/a-y5376e.pdf

[47] Mueller, N., et al. 2012. Closing yield gaps through nutrient and water management. Nature 490: 254-257.
[48] http://data.naturalcapitalproject.org/invest_crop_production/
[49] Macedo, I.C., 1998. Greenhouse gas emissions and energy balances in bio-ethanol production and utilization in Brazil (1996). Biomass and Bioenergy 14, 77–81.
[50] Macedo, I.C., Seabra, J.E.A., Silva, J.E.A.R., 2008. Greenhouse gases emissions in the production and use of ethanol from sugarcane in Brazil: The 2005/2006 averages and a prediction for 2020. Biomass and Bioenergy 32, 582–595.
[51] IFA, 2006. World Fertiliser Use Manual. http://www.fertilizer.org/ifa
[52] Grassini, P., Specht, J.E., Tollenaar, M., Cassman, K.G., 2015. Chapter 2. High-yield maize–soybean cropping systems in the US Corn Belt, in: Crop Physiology. Elsevier Inc., pp. 17–41.
[53] ISU, 2015. Nitrogen use in Iowa Corn Production. Iowa State University. Crop 3073. Available at: https://store.extension.iastate.edu/Product/crop3073-pdf (last accessed: 3/2/2016)
[54] Garrett, J.D., 2012. Concentrations, loads, and yields of select constituents from major tributaries of the Mississippi and Missouri Rivers in Iowa, water years 2004–2008. U.S. Geol Surv Sci Investig Rep 2012–5240 61.
[55] Fonseca, B.M., de Mendonça-Galvão, L., Padovesi-Fonseca, C., de Abreu, L.M., Fernandes, A.C.M., 2014. Nutrient baselines of Cerrado low-order streams: comparing natural and impacted sites in Central Brazil. Environ Monit Assess 186, 19–33.
[56] Mayorga, E., Seitzinger, S.P., Harrison, J.A., Dumont, E., Beusen, A.H.W., Bouwman, A.F., Fekete, B.M., Kroeze, C., Van Drecht, G., 2010. Global Nutrient Export from WaterSheds 2 (NEWS 2): Model development and implementation. Environ. Model. Softw. 25, 837–853.
[57] Doll, P., and S. Siebert. 2002. Global modeling of irrigation water requirements. Water Resources Research 38:1–10.
[58] Siebert, S., Henrich, V., Frenken, K., Burke, J., 2013. Update of the Global Map of Irrigation Areas to version 5. Project report. Available at: http://www.fao.org/nr/water/aquastat/irrigationmap/index60.stm
[59] Frenken, K., Gillet, V., 2010. Aquastat - Irrigation water requirement and water withdrawal by country. FAO. Available at: http://www.fao.org/nr/water/aquastat/water_use_agr/index.stm
[60] Sharp, et al. 2015. InVEST 3.0 User's Guide. Available at: http://data.naturalcapitalproject.org/nightly-build/invest-users-guide/html/.
[61] Gassert, F., Landis, M., Luck, M., Reig, P., Shiao, T., 2013. Aqueduct global maps 2.0. Washington, DC: World Resources Institute. Available online at: http://www.wri.org/publication/aqueduct-metadata-global.
[62]http://waterdata.usgs.gov/ia/nwis and http://water.usgs.gov/watuse/
[63] Hernandes, T.A.D., Bufon, V.B., Seabra, J.E.A., 2013. Water footprint of biofuels in Brazil: assessing regional differences. Biofuels, Bioprod. Biorefining 246–256. This range represents the water withdrawal for sugarcane
[64] Shapouri, Hosein, James A. Duffield, and Michael Q. Wang. 2002. The energy balance of corn ethanol: an update. No. 34075. United States Department of Agriculture, Economic Research Service
[65] Sharp et al. 2015. InVEST 3.2.0 User's Guide for the Sediment Delivery Ratio model. The Natural Capital Project. Available at: http://data.naturalcapitalproject.org/nightly-build/invest-users-guide/html/sdr.html
[66] Roose, 1996. Land husbandry - Components and strategy. Soils bulletin 70. Rome, Italy. Cf. http://www.fao.org/docrep/t1765e/t1765e0e.htm
[67] Hamel, P., R. Chaplin-Kramer, S. Sim, and C. Mueller. 2015. A new approach to modeling the sediment retention service (InVEST 3.0): Case study of the Cape Fear catchment, North Carolina, USA. Science of The Total Environment 524:166–177.
[68] Chaplin-Kramer, R., P. Hamel, R. Sharp, G. Kowal, S. Wolny, S. Sim, and C. Mueller. (n.d.). Landscape configuration is the primary driver of impacts on water quality associated with agricultural expansion. Environmental Research Letters.
[69] BQART is a global regression model (named for its equation, a simple product of B, Q, A, R and T factors) developed to compare regional estimates and avoid simulation discontinuities when using regional models. Cohen, S., A. J. Kettner, J. P. M. Syvitski, and B. M. Fekete. 2013. WBMsed, a distributed global-scale riverine sediment flux model: Model description and validation. Computers & Geosciences 53:80–93.
[70] Alvarez-Cobelas, M., Angeler, D.G., Sanchez-Carrillo, S., 2008. Export of nitrogen from catchments: A worldwide analysis. Environ. Pollut. 156, 261–269.
de Vente, J., Poesen, J., 2005. Predicting soil erosion and sediment yield at the basin scale: Scale issues and semi-quantitative models. Earth-Science Rev. 71, 95–125.
[71] Sharp et al. 2015. InVEST 3.2.0 User's Guide for the GLOBIO Model. The Natural Capital Project. Available at: http://data.naturalcapitalproject.org/nightly-build/invest-users-guide/html/globio.html
[72] Ramankutty, N., et al. 2008. Farming the planet: 1. Geographic distribution of global agricultural lands in the year 2000. Global Biogeochemical Cycles, Vol. 22, GB1003

[73] Ramankutty, N. and J.A. Foley. 2010. ISLSCP II Potential Natural Vegetation Cover. In Hall, Forest G., G. Collatz, B. Meeson, S. Los, E. Brown de Colstoun, and D. Landis (eds.). ISLSCP Initiative II Collection. Data set. Available on-line [http://daac.ornl.gov/] from Oak Ridge National Laboratory Distributed Active Archive Center, Oak Ridge, Tennessee, U.S.A. doi:10.3334/ORNLDAAC/961

[74] Foley, J.A., et al. 2011. Solutions for a cultivated planet. Nature 478: 337-342.

[75] Alkemade et al. 2009. GLOBIO3: a framework to investigate options for reducing global terrestrial biodiversity loss. Ecosystems 12, no. 3: 374-390

[76] Nemecek, T., Heil, A., Huguenin, O., Meier, S., Erzinger, S., Blaser, S., Dux, D., Zimmermann, A., 2004. Life cycle inventories of agricultural production systems - ecoinvent data v1.1. Swiss Centre for Life Cycle Inventories, Dübendorf.

[77] Stewart, L.K., Charlesworth, P.B., Bristow, K.L., 2003. Estimating nitrate leaching under a sugarcane crop using APSIM-SWIM., in: Post, D.A. (Ed.), MODSIM 2003 International Congress on Modelling and Simulation. Volume 2. Modelling and Simulation Society of Australia and New Zealand, pp. 218–223.

[78] Randall, G.W., Vetsch, J.A., Huffman, J.R., 2003. Nitrate losses in subsurface drainage from a corn–soybean rotation as affected by time of nitrogen application and use of nitrapyrin. J. Environ. Qual. 32, 1764–1772.

[79] http://www.waterfootprint.org/Reports/Report47-WaterFootprintCrops-Vol1.pdf

[80] Monfreda, C., Ramankutty, N., Foley, J.A., 2008. Farming the planet: 2. Geographic distribution of crop areas, yields, physiological types, and net primary production in the year 2000. Global Biogeochem. Cycles 22, n/a–n/a.

[81] Reynolds, C.A., Yitayew, M., Slack, D.C., Hutchinson, C.F., Huete, A. and Petersen, M.S. (2000) Estimating crop yields and production by integrating the FAO Crop Specific Water Balance model with real-time satellite data and ground-based ancillary data. International Journal of Remote Sensing (21) 18

[82] Beck, T., Boss, U., Wittstock, B., Baitz, M., Fischer, M. and Sedlbauer, K., 2010. LANCA©. Land use indicator value calculation in life cycle assessment. University of Stuttgart, Germany.

[83] European Commission Joint Research Centre & Institute for Environment and Sustainability (2010) ILCD Handbook. General Guide for Life Cycle Assessment – Detailed Guidance. p71-72. doi:10.2788/38479 Available at: http://publications.jrc.ec.europa.eu/repository/bitstream/JRC48157/ilcd_handbook-general_guide_for_lca-detailed_guidance_12march2010_isbn_fin.pdf

[84] USDA-NASS, 2015. National Agricultural Statistics Services QuickStats Ad-hoc Query Tool. Available at: http://quickstats.nass.usda.gov

[85] Soler, L.S., Verburg, P.H. and Alves, D.S., 2014. Evolution of land use in the Brazilian Amazon: From frontier expansion to market chain dynamics. Land, 3(3), pp.981-1014.

[86] EC(2010) 3751: COMMISSION DECISION of 10 June 2010 on guidelines for the calculation of land carbon stocks for the purpose of Annex V to Directive 2009/28/EC, Official Journal of the European Union, L 151/19.

[87] Ruesch, Aaron, and Holly K. Gibbs. 2008. New IPCC Tier-1 Global Biomass Carbon Map for the Year 2000. Available online: Carbon Dioxide Information Analysis Center [http://cdiac.ornl.gov/], Oak Ridge National Laboratory, Tennessee. Calculations for Table S1a = 300 x .47 x .37; for Table S1b = 220 x .47 x .24; for Table S1ab = average of (52.17, 24.82).

[88] Qui & Turner: in Table S2 of Suppl. Material. Qiu, J., and M. G. Turner. 2013. Spatial interactions among ecosystem services in an urbanizing agricultural watershed. Proceedings of the National Academy of Sciences 110:12149-12154.

[89] IPCC Table S2b, Grassland: IPCC 2006 Guidelines, Voiume 6, Table S6.4. All values in tonne C/ha, converted to carbon based on 47% carbon content of dry matter biomass (see for example section 6.2.1.4 of IPCC 2006).

[90] Renard (1995) Assessing conservation tillage with RUSLE. NCTD.

[91] Killebrew and Wolff (2010). Environmental Impacts of Agricultural Technologies. EPAR Brief 65

[92] Wu and Babcock (1999) Metamodeling potential nitrate water pollution in the Central United States. Journal of Environmental Quality; Nov/Dec 1999; 28, 6

[93] http://apps.cdfa.ca.gov/frep/docs/Corn.html

[94] Prove et al. (1995). Nature and magnitude of soil erosion in sugarcane land on the wet tropical coast of north-eastern Queensland. CSIRO Publishing

[95] Hunsingi (1993). Production of sugarcane. Springer-Verlag Berlin Heidelberg

[96] Hartemink (2008). Advances in Agronomy. Ch. 3: Sugar for Bioethanol: soil and environmental issues. Elsevier.

[97] Thorburn, P.J., Biggs, J.S., Attard, S.J., Kemei, J., 2011. Environmental impacts of irrigated sugarcane production: Nitrogen lost through runoff and leaching. Agric. Ecosyst. Environ. 144, 1–12.

[98] Boulay A-M, Bare J, Benini L, Berger M, Lathuillière M J, Manzardo A, Margni M, Motoshita M, Núñez M, Pastor A V, Ridoutt B, Oki T, Worbe S and Pfister S (*In press*). The WULCA consensus characterization model for water scarcity footprints: Assessing impacts of water consumption based on available water remaining (AWARE). Environ. Sci. Technol.
